# Developmental programming: adverse sexually dimorphic transcriptional programming of gestational testosterone excess in cardiac left ventricle of fetal sheep

**DOI:** 10.1038/s41598-023-29212-9

**Published:** 2023-02-15

**Authors:** Venkateswaran Ramamoorthi Elangovan, Nadia Saadat, Adel Ghnenis, Vasantha Padmanabhan, Arpita K. Vyas

**Affiliations:** 1 Department of Pediatrics, University of Michigan, Ann Arbor, MI USA; 2 College of Medicine, California Northstate University, Elk Grove, CA USA; 3 Department of Pediatrics, Division of Pediatric Endocrinology, School of Medicine, Washington University, St Louis, MO USA

**Keywords:** Disease model, Transcriptomics, Non-coding RNAs, Cardiovascular biology

## Abstract

Adverse in-utero insults during fetal life alters offspring’s developmental trajectory, including that of the cardiovascular system. Gestational hyperandrogenism is once such adverse in-utero insult. Gestational testosterone (T)-treatment, an environment of gestational hyperandrogenism, manifests as hypertension and pathological left ventricular (LV) remodeling in adult ovine offspring. Furthermore, sexual dimorphism is noted in cardiomyocyte number and morphology in fetal life and at birth. This study investigated transcriptional changes and potential biomarkers of prenatal T excess-induced adverse cardiac programming. Genome-wide coding and non-coding (nc) RNA expression were compared between prenatal T-treated (T propionate 100 mg intramuscular twice weekly from days 30 to 90 of gestation; Term: 147 days) and control ovine LV at day 90 fetus in both sexes. Prenatal T induced differential expression of mRNAs in the LV of female (2 down, 5 up) and male (3 down, 1 up) (FDR < 0.05, absolute log2 fold change > 0.5); pathways analysis demonstrated 205 pathways unique to the female, 382 unique to the male and 23 common pathways. In the male, analysis of ncRNA showed differential regulation of 15 lncRNAs (14 down, 1 up) and 27 snoRNAs (26 down and 1 up). These findings suggest sexual dimorphic modulation of cardiac coding and ncRNA with gestational T excess.

## Introduction

Epidemiological studies have determined that the foundation for adult chronic diseases is established early in life^1–3^. In-fact, organisms experiencing an adverse intrauterine environment during a critical period of development have altered molecular and gene expression profiles, reduced ability to adapt to insult and increased susceptibility to adult disease. For instance, susceptibility to cardiovascular disease (CVD) has been strongly linked to adverse in-utero environment^1–4^. Causal evidence in support of developmental origin of pathological cardiac remodeling comes from animal studies^5–12^. Some well-known examples of adverse programming of the cardiovascular system from adverse in-utero environment include pathological left ventricular hypertrophy (LVH)^8,13–19^, reduced cardiomyocyte numbers at birth, hypertension and cardiac dysfunction^20^. Interestingly, many such insults have been found to be associated with androgen excess^21,22^ suggestive of maternal androgen excess having the potential to program cardiovascular dysfunctions in offspring. In support of this premise a recent epidemiological study found elevated third trimester androgen levels to be positively associated with metabolic syndrome including 4.84-fold-increased risk of hypertension in female offspring^23^. Additional support for adverse cardiovascular programming from androgen excess comes from rodent studies that report hypertension during adulthood in offspring exposed prenatally to testosterone (T) excess, which is attenuated by androgen receptor antagonist^24,25^. Our studies in female sheep, a precocial model of translational relevance, provide additional support for adverse cardiovascular programming by prenatal T excess, that are manifested as myocardial disarray, hypertrophy, increase in markers of myocardial stress and hypertension in adulthood^26,27^. More recently impact of prenatal T excess on the myocardium has been reported in both fetal life and at birth^28,29^. Our recent studies found that prenatal T excess from days 30 to 90 days of fetal life increases cardiomyocyte number in female fetuses but not in male fetuses at day 90 of fetal life, although markers of cardiac stress were elevated in both sexes^29^. Another study found early gestational T excess from day 30–60 of gestation reduced cardiomyocyte proliferation in day 135 fetuses, with males impacted more than females^28^. These findings documenting sexually dimorphic T programming of the cardiovascular system are of translational relevance considering the sexual dimorphism in CVD outcomes in human studies^30,31^. However, the mechanisms underlying such sex-specific programming remain to be determined.

Genome-wide transcriptome analysis aids in delineating important pathways involved in organ-specific programming that contributes to functional outcomes. In this regard, our group recently reported that prenatal T excess downregulated mitochondrial, lipid catabolism, and PPAR signaling genes in the liver and dysregulated mitochondrial and ncRNA gene pathways in muscle, consistent with the lipotoxic and insulin-resistant hepatic and muscle phenotype of these animals^32^. Of importance, these changes were accompanied by tissue-specific changes in noncoding (nc) RNA^32^ that have been implicated in several disease processes, including hyperandrogenic disorders such as PCOS^33,34^, the model the prenatal T-treated sheep mimic^35^. In the context of our current study, data is emerging regarding the contribution of ncRNA dysregulation in CVD^36^, as novel biomarkers of myocardial disease and as potential therapeutics^37^. As such, epigenetic mechanisms that include changes in ncRNAs and their link to gene transcriptional changes are important considerations for relating to postnatal functional outcomes, identifying biomarkers, and targeting intervention strategies. Therefore, the objectives of our current study are to determine (1) sex differences in coding RNA and ncRNA in left ventricle (LV) of day 90 fetuses; (2) sex-specific changes in gene pathways affected by gestational T excess, (3) the relationship between changes in ncRNA with coding RNA and (4) if sexually dimorphic changes in ncRNA and coding RNA would explain the sexually dimorphic cardiomyocyte phenotype.

## Results

### Descriptive statistics

Post-trimming, samples across both total RNA and noncoding RNA exhibited high mean read quality scores, per sequence GC content ranging from 20 to 80% and sequence duplication levels upto 30% (Supplementary Figures S1 and S2).

### Sex-specific coding RNA expression

**Figure 1:**
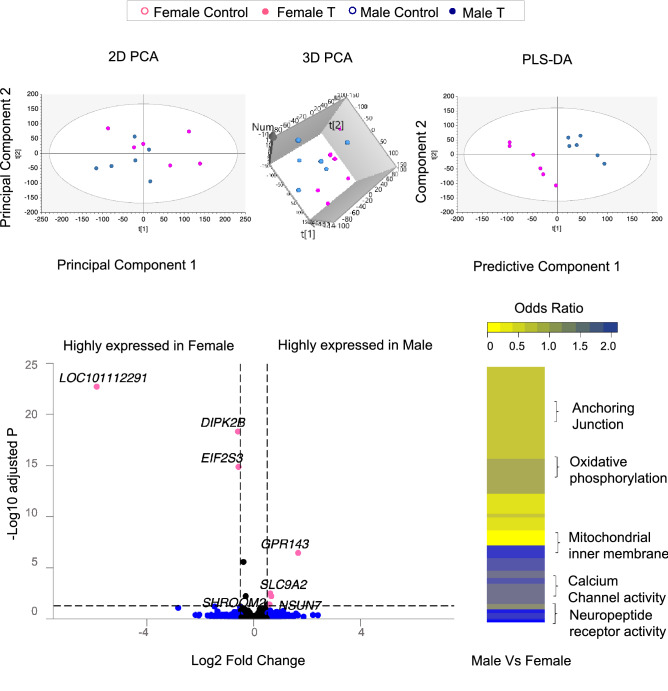
Total RNA sample clustering and tissue-specific enrichment in control animals. Principal Component Analysis (PCA) 2D from fetal cardiac tissue of control animals are shown on the top. For the PCA the 2D plots, 3D PCA plots are plotted with principal component 1 on X-axis and principal component 2 on Y-axis and PLS-DA plots are plotted with component 1 on X-axis and component 2 on Y-axis showing separation between the Female (dark pink circle) and Male (blue circle) from control animals. Each point represents one animal. The Volcano plot of differential gene expression comparing female and male fetal cardiac tissues in control animals are shown on the bottom-left. Genes are plotted by log2 fold change and − log10 adjusted *p*-values. The pink points represent genes that have absolute log2 fold change > 0.5 and FDR < 0.05. Blue dots represent those that met absolute log2FC > 0.5 but did not meet − log10 adjusted p and black dots represent those that did not meet log2FC or − log10adjust p. Heatmap of differentially regulated gene pathways across both female and male at false discovery rate < 0.01 are shown on the bottom-right.

A partial separation in coding RNA transcriptome profiles was observed in 2D and 3D PCA plots generated using unsupervised model. However, PLS-DA (Partial Least Squares Discriminant analysis) based supervised model (with groups as an outcome variable) demonstrated a clear separation of male and female fetal LV transcriptome profiles (Fig. 1). Comparison of gene expression (FDR < 0.05 and absolute log2 fold change (abs log2FC) > 0.5) between female and male LV controls demonstrated 2 genes specific for female and 4 genes specific for the male (Fig. 1, Table 1 listed with chromosomal locations, Supplemental Table S1). The genes with higher expression in the female were the coding genes for *DIPK2B* (divergent protein kinase domain 2B) and *EIF2S3* (eukaryotic translation initiation factor 2 subunit gamma). Similarly, in the male, there was higher expression of *GPR143* (G protein—coupled receptor 143), *NSUN7* (NOP2/Sun RNA methyltransferase family number 7), *SLC9A2* (solute carrier family 9 member A2) and *SHROOM2* (shroom family member 2).

**Table 1:** Top differentially expressed coding RNA between the control female and control male fetal LV tissue.

Female					Male				
Gene	log2FC	padj	Chr	Location	Gene	lof2FC	padj	Chr	Location
*LOC101112291*	− 5.879	0.000	X	NC_040278.1 (76108652..76145778)	*GPR143*	1.663	0	X	NC_040278.1 (6976011..6999540)
*DIPK2B*	− 0.591	0.000	X	NC_040278.1 (45748914..45789765 complement)	*NSUN7*	0.657	0.006	6	NC_040257.1 (65217328..65265711)
*EIF2S3*	− 0.57	0.000	X	NC_040278.1 (23702311..23717599)	*SLC9A2*	0.619	0.003	3	NC_040254.1 (105508785..105597899 complement)
					*SHROOM2*	0.588	0.038	X	NC_040278.1 (6803408..6949609 complement)

Genes in either the female or male control sheep enriched at adjusted-p value (padj) < 0.05 and absolute log2 fold change (log2FC) > 0.5 are represented. Genes highly expressed in the female fetal LV tissue are represented as negative values and those highly expressed in the male fetal LV tissue are represented as positive values. ncRNA is highlighted in grey.

### Sex-specific pathways in control animals

Analysis of pathways differentially modulated between the female and male cardiac tissue in control animals revealed 139 dysregulated pathways (FDR < 0.01) (Fig. 1; Supplemental Table S2) out of which 43 enriched for males and 96 enriched for the females.

### Sex-specific differences in ncRNA expression

Comparison of ncRNA expression (FDR < 0.05 and absolute log2 fold change (abs log2FC) > 0.5) in female and male LV controls revealed only one lncRNA *LOC101112291* (uncharacterized) was upregulated in females. This lncRNA *LOC101112291* (uncharacterized) also known as Xist is highly expressed in females and the primary role for this gene is regulating X-chromosome inactivation (Table 2 listed with chromosomal locations, Supplemental Table S3).

**Table 2:** ncRNA differentially expressed between the control female and control male fetal LV tissue.

Gene	log2FC	padj	Chr	Location
LOC101112291	− 5.809	3.02E − 15	X	NC_040278.1 (76108652..76145778)

ncRNA genes in the female control compared to the male control and enriched at adjusted-p value (padj) < 0.05 and absolute log2 fold change (log2FC) > 0.5 are represented. Genes highly expressed in the female fetal LV tissue are represented as negative values. Among all classes only one lncRNA was differentially expressed in the female fetal LV tissue compared to the male fetal LV tissue.

### Potential sex-specific RNA biomarkers in control

The top 30 coding RNA PLS-DA VIP gene comparing female and male control LVs are listed in Table 3 along with their chromosomal locations. Among those with high VIP values, *LOC101112291, DIPK2B, GPR143, EIF2S3, SLC9A2, SHROOM2, KDM6A,* were confirmed as having the potential to serve as biomarkers since they also met the padj < 0.05 significance from the differential expression analysis*.*

**Table 3:** Top 30 sex-specific differences in potential coding biomarkers in LV tissue of control sheep.

ID	VIP	log2FC	padj	chr	Location
*LOC114110268*	2.853	0.349	0.581	22	NC_040273.1 (4201624..4203403 complement)
*LOC101112291*	2.853	− 5.879	0	X	NC_040278.1 (76108652..76145778)
*DIPK2B*	2.751	− 0.591	0	X	NC_040278.1 (45748914..45789765 complement)
*MED14*	2.677	− 0.129	0.382	X	NC_040278.1 (41544083..41606772 complement)
*GPR143*	2.656	1.663	0	X	NC_040278.1 (6976011..6999540)
*EIF2S3*	2.555	− 0.57	0	X	NC_040278.1 (23702311..23717599)
*DNAH6*	2.489	− 3.27	NA	3	NC_040254.1 (60391449..60677762)
*TAF1A*	2.477	0.365	0.517	12	NC_040263.1 (28357524..28388826 complement)
*SLC9A2*	2.466	0.619	0.003	3	NC_040254.1 (105508785..105597899 complement)
*SHROOM2*	2.442	0.588	0.038	X	NC_040278.1 (6803408..6949609 complement)
*KDM6A*	2.425	− 0.385	0	X	NC_040278.1 (45535702..45717259)
*LOC114109659*	2.424	1.415	NA	20	NC_040271.1 (35149285..35150694)
*ATP6V1E2*	2.422	− 0.194	0.787	3	NC_040254.1 (83038084..83062396)
*SCT*	2.414	− 0.382	0.442	21	NC_040272.1 (52395939..52397470)
*TRNAE-UUC_4*	2.408	0.558	NA	11	NC_056064.1 (24587024..24587097)
*RCC1*	2.399	− 0.277	0.258	2	NC_040253.1 (254401876..254419515 complement)
*LOC101118635*	2.395	1.099	0.163	14	NC_040265.1 (70653287..70675125)
*CDCP1*	2.391	− 0.596	0.299	19	NC_040270.1 (56011499..56073191)
*LOC114115129*	2.39	0.694	0.442	5	NC_040256.1 (46591790..46591896 complement)
*LOC114118727*	2.385	0.431	0.374	16	NC_040267.1 (8494942..8512073 complement)
*TP53*	2.384	− 0.173	0.329	11	NC_040262.1 (36037234..36050138)
*LOC101112843*	2.36	− 0.722	0.208	14	NC_040265.1 (65906556..65918271 complement)
*LOC106991047*	2.355	1.217	NA	3	NC_040254.1 (43329306..43498366 complement)
*TBL1X*	2.353	0.194	0.382	X	NC_040278.1 (7004107..7077329 complement)
*LOC106991067*	2.336	0.272	NA	3	NC_040254.1 (106412531..106413623)
*LOC101103215*	2.335	− 0.494	0.061	2	NC_040253.1 (260329990..260334627 complement)
*TRNAW-CCA_63*	2.334	− 0.829	NA		
*LOC114109776*	2.321	NA	NA	20	NC_040271.1 (44516538..44516644)
*POLR3A*	2.318	− 0.074	0.545	25	NC_040276.1 (34875438..34923303 complement)
*MAP1S*	2.316	− 0.17	0.245	5	NC_040256.1 (5409141..5440949 complement)

Top 30 Coding RNA biomarkers comparing control female and male-fetal cardiac tissue based on variable importance in Projection values (VIP) along with the corresponding log2FC and padj values obtained from DESeq2 analysis for the same determinants are represented. ncRNA is highlighted in grey.

### Sex-specific impact of gestational T-treatment on LV gene expression

#### Female

**Figure 2:**
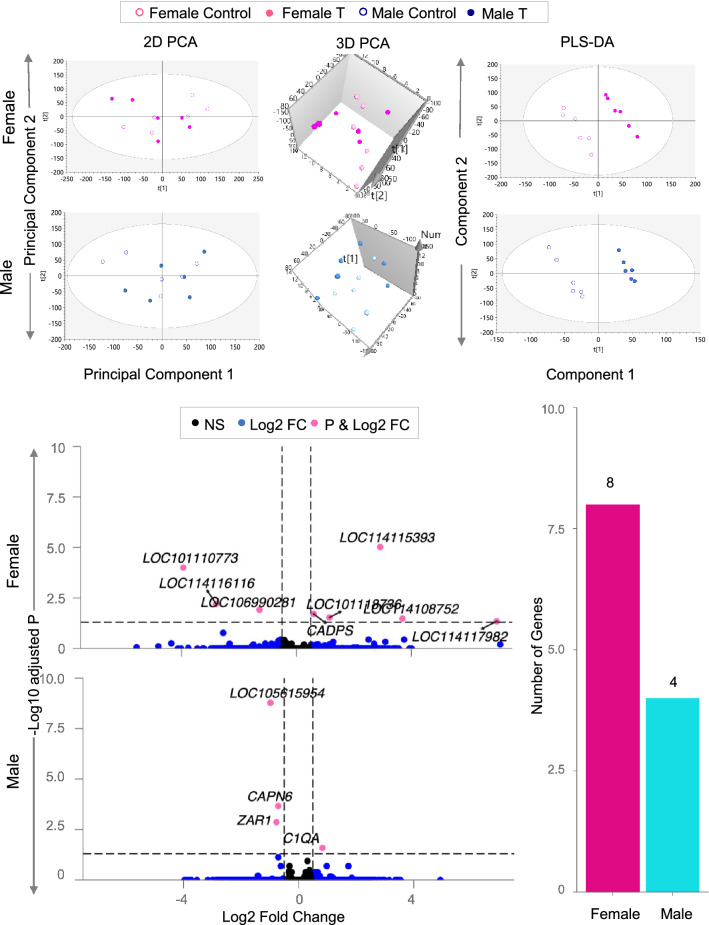
Total RNA Sample Clustering and Differential Expression in Female and Male from Prenatal T-Treated Animals. The 2D plots showing clustering between control (dark pink open circle for female and blue open circle for male) and prenatal T-treated fetal cardiac tissue (dark pink closed circle for female or blue closed circle for male) groups in the coding RNA from fetal cardiac tissue are shown on the top. For the PCA the 2D, 3D PCA plots are plotted with principal component 1 on X-axis and principal component 2 on Y-axis and PLS-DA plots are plotted with component 1 on X-axis and component 2 on Y-axis, with each point representing one animal. The Volcano plot showing differential gene expression in the female or male fetal cardiac tissue comparing prenatal T-treated animals against control animals are shown on the bottom-left. Genes are plotted by log2 fold change in the X-axis and − log10 p-adjusted values in the Y-axis. Pink points denote the genes that have absolute log2 foldchange > 0.5 and p-adjusted values < 0.05. Black dots represent genes that did not meet p-adjusted cut-off of < 0.05 or absolute log2 fold change > 0.5, and blue dots represent genes which met the absolute log2 fold change > 0.5 but did not meet p-adjusted cut-off of < 0.05. The bar plots on the bottom-right represents the number of genes differentially modulated that are unique to either female(pink) or male(teal).

The 2D PCA score plot and the 3D score plots showed an overlap between the control and T-treated groups (Fig. 2). Further examination using PLS-DA that accounted for the variance between the two groups showed clear separation between the control and T-treated groups (Fig. 2). Gestational T-treatment induced differential expression of 8 genes in the LV of female fetus with 5 upregulated and 3 downregulated genes at FDR < 0.05 and absolute log2FC > 0.5. The upregulated genes include *LOC114117982* (F-box only protein 27-like*), LOC114108752 (*sentrin-specific protease 6-like)*, LOC114115393* (probable phospholipid-transporting ATPase VD)*, LOC101118736 (*HIG1 domain family member 1A, mitochondrial)*, CADPS* (calcium dependent secretion activator) and the downregulated genes include *LOC106990281* (atypical chemokine receptor 2-like), LOC114116116 (UL16-binding protein 1-like), *LOC101110773* (elongation factor 1-alpha 1) (Fig. 2, Table 4 listed with chromosomal locations, Supplemental Table S4).

**Table 4:** Gestational T modulated coding RNA in the female and male fetal LV tissue.

Female					Male				
Gene	log2FC	padj	chr	Location	Gene	log2FC	padj	Chr	Location
*LOC114117982*	6.965	0.045	14	NC_040265.1 (51382972..51386724 complement)	*C1QA*	0.831	0.025	2	NC_040253.1 (259717526..259720581 complement)
*LOC114108752*	3.687	0.033	8	NC_040259.1 (2701669..2714773)	*CAPN6*	− 0.713	0	X	NC_056080.1 (123749062..123773801)
*LOC114115393*	2.916	0	6	NC_040257.1 (20115067..20131231 complement)	*ZAR1*	− 0.774	0.001	6	NC_040257.1 (73764051..73768165)
*LOC101118736*	1.153	0.029	7	NC_040258.1 (88835036..88836333 complement)	*LOC105615954*	− 0.982	0	8	NC_040259.1 (28057565..28059544 complement)
*CADPS*	0.594	0.019	19	NC_040270.1 (40228266..40719771)					
*LOC106990281*	− 1.285	0.012	19	NC_040270.1 (15877303..15881725)					
*LOC114116116*	− 2.791	0.006	8	NC_040259.1 (81251549..81266689)					
*LOC101110773*	− 3.935	0	10	NC_040261.1 (30962028..30963797)					

Genes highly modulated in either the female or male fetal LV tissue with adjusted p-value (padj) < 0.05 and absolute log2 fold change(log2FC) > 0.5 are represented. The top genes sorted by absolute magnitude are represented with their direction of effect.

#### Male

Similar to the score plots from female fetal LV the 2D PCA and 3D PCA plots showed overlap between control and T treated groups. However, the separation became more distinct in the PLS-DA model (Fig. 2). The number of genes differentially expressed in the male LV were fewer than in the female with 4 differentially expressed genes, which included 1 upregulated and 3 downregulated at FDR < 0.05 and absolute log2FC > 0.5. The only upregulated gene was *C1QA* (Complement C1q A Chain)*,* and the downregulated genes were *CAPN6* (Calpain 6), *ZAR1* (Zygote Arrest 1) *and LOC105615954 (*uncharacterized) (Fig. 2, Table 4 listed with chromosomal locations, Supplemental Table S5).

Gestational T-treatment induced dysregulated genes were unique for female and male LV tissue.

### Sex differences in pathways enriched by gestational T treatment

**Figure 3:**
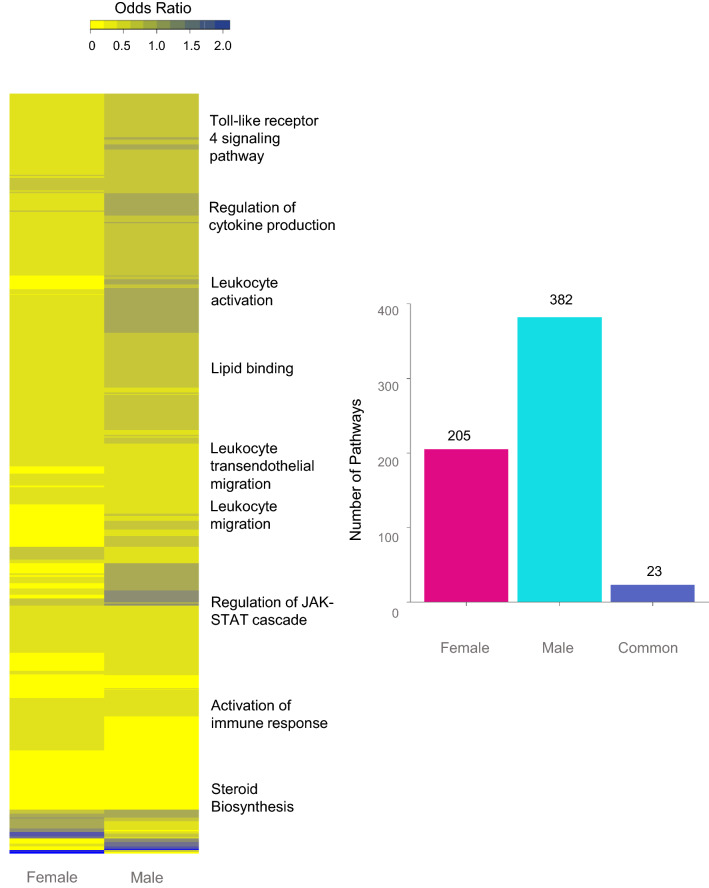
Functional Enrichment of Pathways Enriched in Female and Male Fetal Cardiac Tissue. Heat map (Left) representing the differentially regulated gene pathways in female and male fetal cardiac tissue from prenatal T-treated animals compared against control animals and enriched at FDR < 0.01. The bar plots (right) represent the number of gene pathways differentially modulated that are unique to female (pink bar), male fetal cardiac tissues (teal bar) or commonly dysregulated in both tissues (dark blue bar).

**Figure 4:**
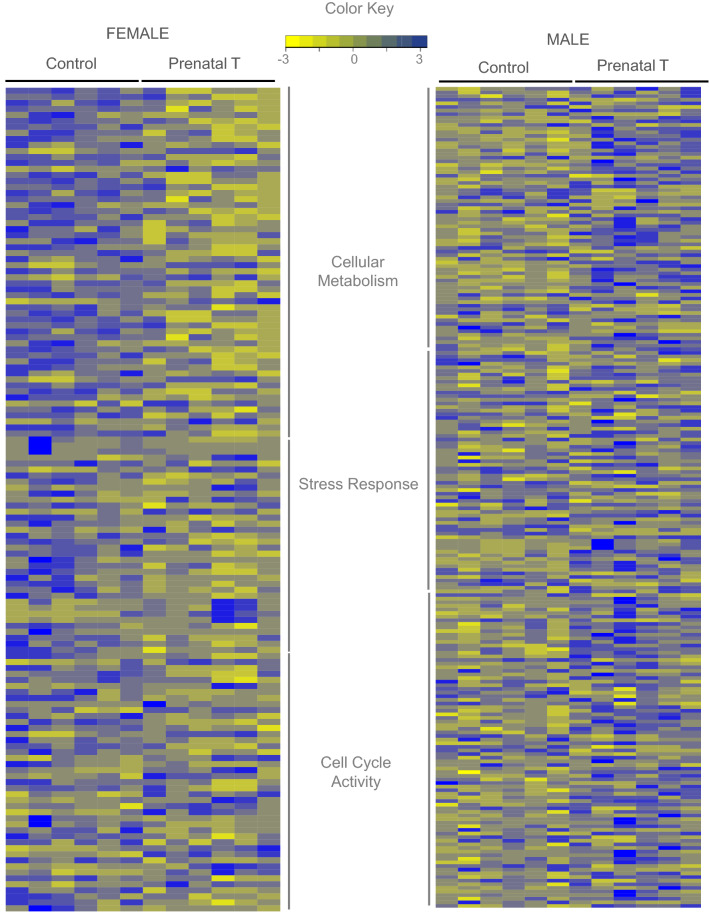
Expression Levels of Genes in Commonly Dysregulated Pathways between Male and Female in Response to prenatal T. Heatmap showing genes involved in the pathways representing metabolism, response to stress and cell cycle activity that is dysregulated in both female (C = 6, T = 6) (left panel) and male (C = 6, T = 6) (right panel). The genes associated with the gene pathway in controls animals and prenatal-T treated animals are plotted along a gradient of colors, with blue representing highest and yellow the lowest normalized counts.

**Figure 5:**
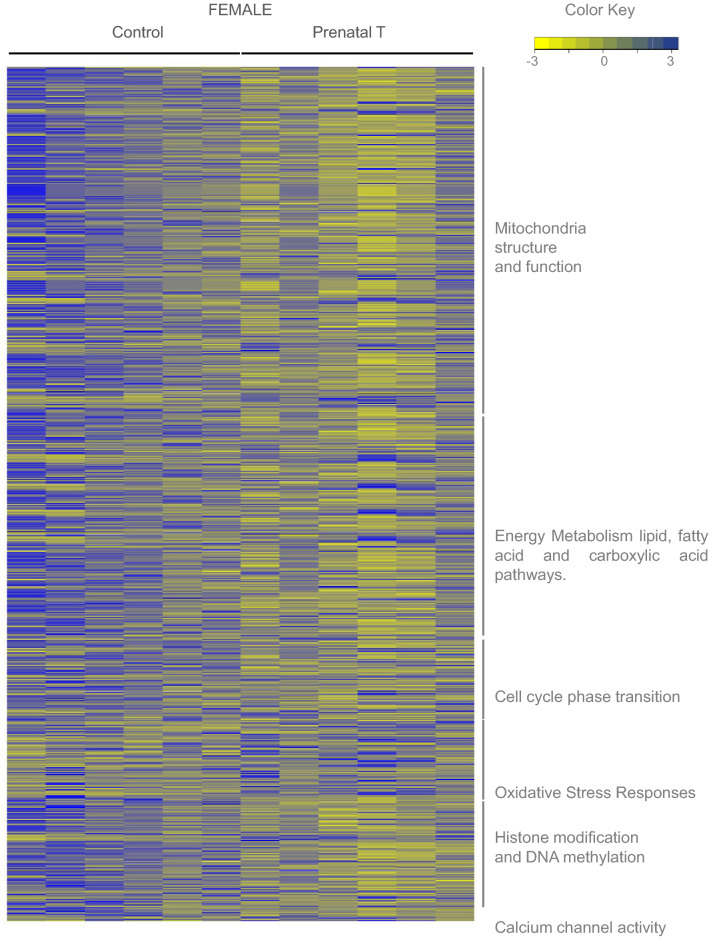
Expression Levels of Genes in Dysregulated Pathways Unique to Female Fetal Cardiac Tissue in Response to prenatal T. Heatmap showing genes involved in the pathways representing selected pathways that is dysregulated uniquely in female (C = 6, T = 6). The genes associated with the gene pathway in controls animals and prenatal-T treated animals are plotted along a gradient of colors, with blue representing highest and yellow the lowest normalized counts.

**Figure 6:**
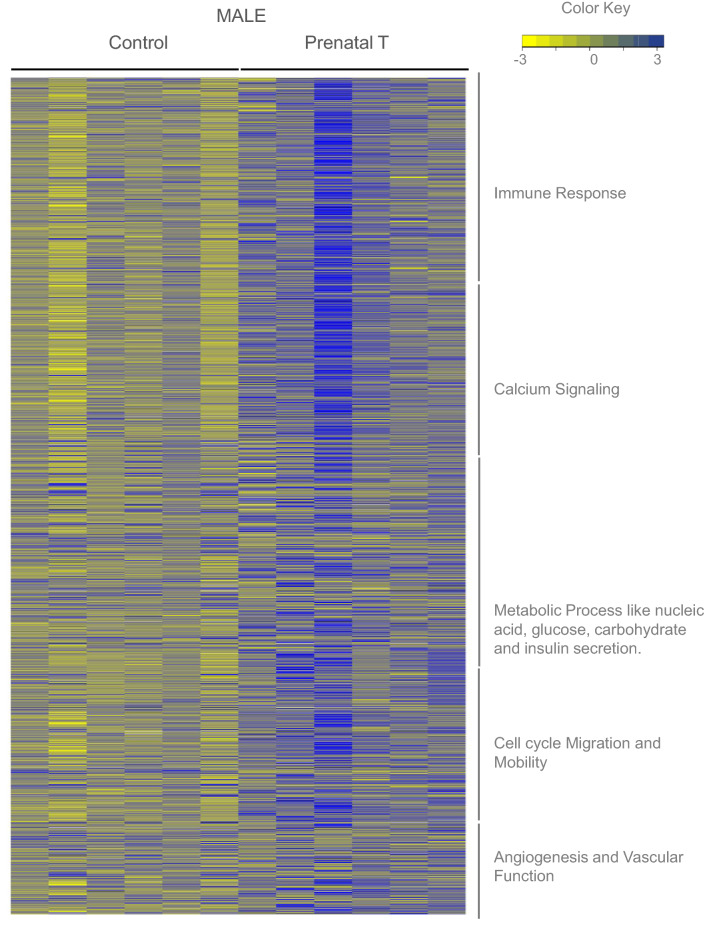
Expression Levels of Genes in Dysregulated Pathways Unique to Male Fetal Cardiac Tissue in Response to prenatal T. Heatmap showing genes involved in the pathways representing selected pathways that is dysregulated uniquely in male (C = 6, T = 6). The genes associated with the gene pathway in controls animals and prenatal-T treated animals are plotted along a gradient of colors, with blue representing highest and yellow the lowest normalized counts.

Gestational T treatment modulated 205 and 382 unique pathways in the female and male respectively, while there were 23 pathways commonly dysregulated in both sexes at FDR < 0.01 (Fig. 3, Supplemental Table S6). Specifically, pathways related to cellular metabolism, stress response and cell-cycle activity were commonly dysregulated in both sexes (Fig. 4, Supplemental Table S7). On the other hand, several pathways pertaining to mitochondrial structure and function, energy metabolism, lipid, fatty acid and carboxylic acid pathways, oxidative stress, cell cycle phase transition, signaling processes like calcium channel activity, and epigenetic dysregulation such histone modification and DNA methylation were enriched uniquely in the female LV tissue (Fig. 5, Supplemental Table S7). In contrast, pathways belonging to immune response, calcium signaling, metabolic processes like nucleic acid, glucose, carbohydrate and insulin secretion, cell cycle migration and those related to vascular function and angiogenesis were uniquely enriched in the male fetal LV tissue (Fig. 6, Supplemental Table S7).

### Potential coding RNA biomarkers of gestational-T treatment

#### Female

The top 30 coding RNA PLS-DA VIP genes having the potential to serve as biomarkers of gestational T-treatment included 15 downregulated and 15 upregulated genes. The genes with high VIP values are listed in Table 5 along with chromosomal locations. Among these with high VIP values, one downregulated uncharacterized gene *LOC106990281* and two upregulated uncharacterized genes *(LOC114115393 and LOC101118736)* also met the padj < 0.05 significance as determined by differential expression analysis, identifying them as strong candidates to serve as potential biomarkers*.*

**Table 5:** Top 30 potential coding biomarkers in female fetal LV exposed to excess T.

ID	VIP	log2FC	padj	Chr	Location
*TNNC2*	2.95	− 0.401	0.998	13	NC_040264.1 (77960813..77963931 complement)
*DNAH6*	2.92	− 2.741	0.998	3	NC_040254.1 (60391449..60677762)
*LOC114116348*	2.92	− 3.241	0.998	1	NC_040252.1 (55945340..55966532)
*LOC114114839*	2.84	− 1.415	0.998	5	NC_040256.1 (17242460..17251151)
*DPY19L2*	2.82	1.265	0.574	4	NC_040255.1 (68692475..68788865)
*LOC101118736*	2.81	1.153	0.029	7	NC_040258.1 (88835036..88836333 complement)
*LOC114115383*	2.81	− 2.013	0.998	6	NC_040257.1 (3367322..3375341 complement)
*LOC114114885*	2.8	− 0.594	0.629	5	NC_040256.1 (54581742..54586904)
*LOC114115393*	2.79	2.916	0	6	NC_040257.1 (20115067..20131231 complement)
*TAF6*	2.79	− 0.136	0.973	24	NC_040275.1 (37014525..37021834)
*IL12A*	2.79	− 2.039	0.998	1	NC_040252.1 (248087948..248094879 complement)
*LOC114108739*	2.77	2.111	0.889	1	NC_040252.1 (70051200..70072788 complement)
*LOC106990219*	2.76	1.434	0.998	1	NC_040252.1 (91548992..91560737)
*GRHL2*	2.76	1.017	0.998	9	NC_040260.1 (83548199..83722850 complement)
*LOC114116509*	2.76	1.996	0.889	9	NC_040260.1 (76671415..76671521)
*LOC105610993*	2.75	− 0.864	0.571	19	NC_040270.1 (15800899..15868556)
*CAMK2N2*	2.75	0.757	0.998	1	NC_040252.1 (221433766..221435945)
*TRNAS-GGA_18*	2.75	1.859	0.998		
*RCC1L*	2.75	− 0.103	0.998	24	NC_040275.1 (33377406..33404451 complement)
*VSIG1*	2.75	0.755	0.998	X	NC_040278.1 (135605002..135637191 complement)
*LOC114111381*	2.73	0.685	0.998	1	NC_040252.1 (218973968..218974036 complement)
*LOC106990281*	2.71	− 1.285	0.012	19	NC_040270.1 (15877303..15881725)
*LOC105604073*	2.71	− 2.267	0.998	21	NC_040272.1 (37886861..37890892 complement)
*TRNAW-CCA_63*	2.71	− 2.411	0.998		
*GPR37*	2.7	− 1.146	0.998	4	NC_040255.1 (97339539..97357705 complement)
*LOC101105505*	2.7	0.604	0.998	7	NC_040258.1 (44712167..44717117 complement)
*AQP6*	2.67	2.023	0.998	3	NC_040254.1 (146235352..146239160 complement)
*NAGA*	2.66	− 0.171	0.571	3	NC_040254.1 (233912361..233922397 complement)
*TUBGCP4*	2.66	0.079	0.998	18	NC_040269.1 (55442666..55485857)
*TRNAE-UUC_4*	2.66	1.216	0.998	4	NC_040255.1 (16147586..16147658)

Top 30 coding RNA biomarkers comparing control female and T-treated female fetal LV tissue based on variable importance in Projection values. (M2.VIP[3]) along with the corresponding log2FC and padj values obtained from DESeq2 analysis for the same determinants are represented. ncRNA is highlighted in grey.

#### Male

The top 30 coding RNA PLS-DA VIP genes having the potential to serve as biomarkers of gestational T-treatment in the male included 12 downregulated and 18 upregulated genes. The genes with high VIP values are listed in Table 6 along with their chromosomal locations. Among the dysregulated genes three genes *ZAR1, LOC105615954 and CAPN6* that were downregulated met the significance threshold of padj < 0.05 as determined by differential expression analysis thus making them strong candidates to serve as potential biomarkers.

**Table 6:** Top 30 potential coding biomarkers in male fetal LV tissue exposed to excess T.

ID	VIP	log2FC	padj	chr	Location
*ZAR1*	3.22	− 0.774	0.001	6	NC_040257.1 (73764051..73768165)
*LOC105615954*	3.21	− 0.982	0	8	NC_040259.1 (28057565..28059544 complement)
*NR1H3*	3.11	0.217	0.61	15	NC_040266.1 (83401617..83414464)
*PDSS2*	3.07	− 0.111	1	8	NC_040259.1 (32952748..33233373)
*GAS1*	3.05	0.308	0.113	2	NC_040253.1 (34500120..34502580)
*UBE2F*	3.03	0.121	1	1	NC_040252.1 (3143028..3188912 complement)
*LOC101113807*	3.02	2.149	1	1	NC_040252.1 (93438741..93448528 complement)
*BYSL*	3.02	0.123	1	20	NC_040271.1 (17440894..17450957)
*LOC114115129*	3	− 0.625	1	5	NC_040256.1 (46591790..46591896 complement)
*MON1A*	2.99	0.187	1	19	NC_040270.1 (52323926..52335056)
*FAM83C*	2.98	− 2.003	1	13	NC_040264.1 (67349627..67356827 complement)
*ATP10D*	2.97	− 0.238	0.401	6	NC_040257.1 (72638753..72767402)
*GDF5*	2.93	− 0.655	1	13	NC_040264.1 (67474035..67479151 complement)
*SYT12*	2.93	0.666	0.401	21	NC_040272.1 (46768944..46791736)
*ABT1*	2.92	− 0.141	1	20	NC_040271.1 (34092796..34108166 complement)
*TTC1*	2.92	0.171	1	5	NC_040256.1 (74531912..74580401)
*TRNAM-CAU_5*	2.9	2.342	1		
*LOC114110 177*	2.89	2.182	1	21	NC_040272.1 (8941268..8941374)
*LOC106991502*	2.89	− 0.455	1	12	NC_040263.1 (83468753..83469440 complement)
*SYT17*	2.88	0.243	0.401	24	NC_040275.1 (16922918..17006171 complement)
*PLAC9*	2.88	0.379	0.315	25	NC_040276.1 (36370845..36382385 complement)
*DHODH*	2.88	0.166	1	14	NC_040265.1 (41892301..41905265 complement)
*NAB1*	2.87	0.172	0.824	2	NC_040253.1 (128345709..128397329 complement)
*LOC114113924*	2.87	− 1.419	0.61	1	NC_040252.1 (38177..39148)
*LOC114110995*	2.87	1.785	1	1	NC_040252.1 (263047828..263047934 complement)
*CAPN6*	2.87	− 0.713	0	X	NC_040278.1 (132176018..132200792)
*LOC101111394*	2.86	− 1.48	1	16	NC_040267.1 (40550255..40755659 complement)
*NXPH3*	2.85	0.43	1	11	NC_040262.1 (26287358..26291570)
*LOC114116100*	2.84	3.15	1	8	NC_040259.1 (62917292..62936188 complement)
*LOC114116498*	2.84	− 1.262	1	9	NC_040260.1 (39016586..39016652 complement)

Top 30 Coding RNA biomarkers comparing control male and T-treated male fetal LV tissue based on variable importance in Projection values. (M2.VIP[3]) along with the corresponding log2FC and padj values obtained from DESeq2 analysis for the same determinants are represented. ncRNA is highlighted in grey.

### Impact of gestational T treatment on ncRNA

#### Female

2D and 3D PCA plots generated after removal of an outlier from lncRNA, snoRNA and snRNA by unsupervised clustering but keeping all samples in miRNA showed overlap between control and prenatal T-treated groups (results prior to removal of the outlier and after removing the outliers are included in the supplement, Figures S3 and S4). However, PLS-DA based supervised model performed demonstrated a clear separation of T-treated and control LV ncRNA expression profiles (Figure S4). Analysis by DESeq2 after removal of the outliers from lncRNA, snoRNA and snRNA and keeping all samples for miRNA (no outliers were observed in miRNA), found no significant effect of gestational T treatment on any ncRNA in differential expression analysis (lncRNA, miRNA, snoRNA, snRNA) (Supplemental Figure S6, Table S8). The potential ncRNA signatures of gestational T excess of female LV based on PLS-DA model VIP values are listed in Supplemental Table S9; none of these met the criteria of FDR < 0.05.

#### Male

**Figure 7:**
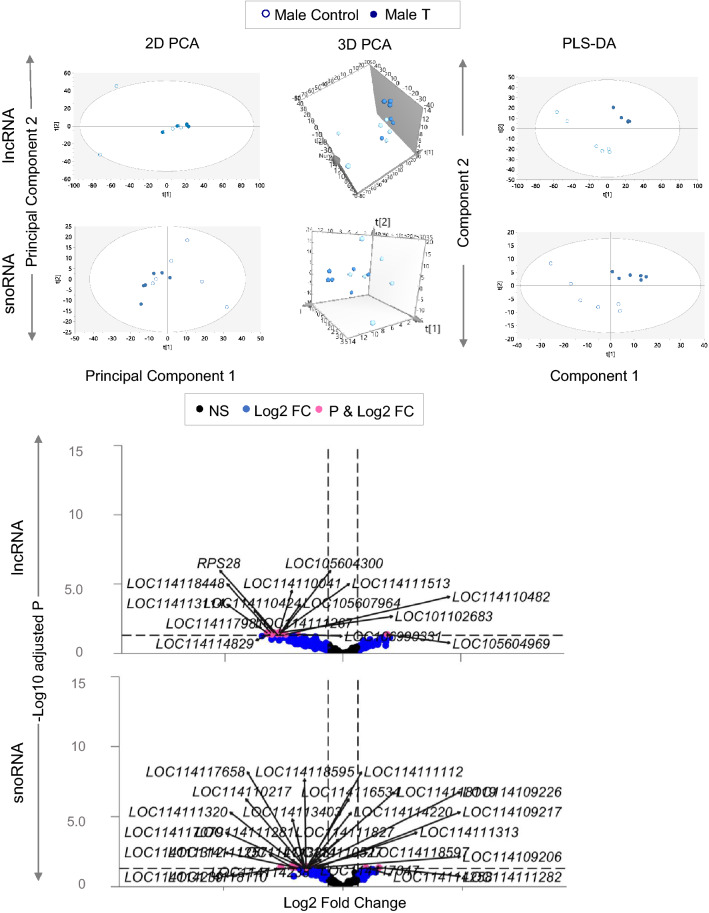
Long non-coding and Small nucleolar RNA Clustering and Differential Expression in males: The 2D, 3D PCA plots and PLS-DA showing clustering between control (blue open circle for male) and prenatal T-treated fetal cardiac tissue (blue closed circle for male) groups in the lncRNA and snoRNA from fetal cardiac tissue are shown on the top. For the PCA the 2D plots, 3D plots are plotted with principal component 1 on X-axis and principal component 2 on Y-axis, and PLS-DA plots are plotted with component 1 in the X-axis and component 2 on the Y-axis, with each point representing one animal. Volcano plots showing differential lncRNA and snoRNA expression in the male cardiac tissues of prenatal T-treated animals compared against the control animals are shown on the bottom. The log2 fold change values are represented against the X-axis and − log10 adjusted *p*-values are represented along the Y-axis. Pink dots represent those ncRNA that met absolute log2FC > 0.5 and p-adjusted cut-off of < 0.05. Black dots represent ncRNA genes that did not meet either p-adjusted cut-off of < 0.05 or absolute log2FC > 0.5 and blue dots represent genes which met absolute log2 fold change > 0.5 but did not meet the adjusted *p*-values < 0.05.

Across all classes of ncRNA studied in the male fetal LV, 2D and 3D PCA plots generated by unsupervised clustering showed an overlap between control and gestational T-treated groups. PLS-DA based supervised model demonstrated a clear separation of T treated vs control fetal LV tissue ncRNA expression profiles (lncRNA and snoRNA—Fig. 7, PCA and PLS DA models of all ncRNA classes are included in Supplemental Figure S5). Gestational T treatment modulated 15 different lncRNAs and 27 different snoRNAs at FDR < 0.05 and absolute log2FC > 0.5. The potential non-coding RNA signatures of T treatment on male fetal LV based on PLS-DA model VIP values are listed in Supplemental Table S10. One of the snoRNA *LOC114114258* which was identified as a potential snoRNA signature for T treatment on male fetal LV was also significantly differentially expressed in DESeq2 analysis.

#### lncRNA

Gestational T treatment induced the expression of one lncRNA (LOC105604969) and reduced the expression of 14 other lncRNAs (Fig. 7, Table 7 listed with chromosomal locations and Supplemental Table S11).

**Table 7:** Top gestational T modulated lncRNA and snoRNA in the male fetuses.

Gene	log2FC	padj	Chr	Location	Gene	log2FC	padj	Chr	Location
*LOC105607964*	− 2.117	0.031	2	NC_040253.1 (76068534..76235875 complement)	*LOC114111320*	− 1.438	0.036	1	NC_040252.1 (14761546..14761680 complement)
*LOC114118448*	− 2.451	0.031	15	NC_040266.1 (68222402..68270216)	*LOC114111257*	− 1.736	0.036	1	NC_040252.1 (278641140..278641275)
*LOC114110041*	− 2.168	0.031	21	NC_040272.1 (51298663..51301230)	*LOC114114220*	− 1.15	0.036	3	NC_040254.1 (185898785..185898920 complement)
*LOC101102683*	− 2.107	0.031	21	NC_040272.1 (51374427..51377030)	*LOC114116534*	− 1.2	0.036	9	NC_040260.1 (92154296..92154426)
*LOC114110424*	− 2.351	0.031	23	NC_040274.1 (38974710..39040905 complement)	*LOC114117047*	− 1.182	0.036	11	NC_040262.1 (35606839..35606974)
*LOC114111513*	− 2.108	0.031	X	NC_040278.1 (7420759..7554518)	*LOC114118110*	− 2.082	0.036	14	NC_040265.1 (59381451..59381537)
*LOC114113114*	− 2.574	0.032	2	NC_040253.1 (142044466..142046716)	*LOC114118595*	− 1.238	0.036	15	NC_040266.1 (38316004..38316093)
*RPS28*	− 2.226	0.043	5	NC_040256.1 (15285478..15286624)	*LOC114109226*	− 1.05	0.036	18	NC_040269.1 (66396221..66396292)
*LOC114117981*	− 2.407	0.043	14	NC_040265.1 (50588602..50718739)	*LOC114111281*	− 1.325	0.043	1	NC_040252.1 (20121922..20122001)
*LOC106990331*	− 1.534	0.043	16	NC_040267.1 (14798747..14913203)	*LOC114111282*	1.204	0.043	1	NC_040252.1 (20123863..20123933)
*LOC114110482*	− 1.938	0.043	23	NC_040274.1 (39993829..40033362)	*LOC114113381*	− 1.324	0.043	2	NC_040253.1 (236079342..236079421)
*LOC105604969*	1.502	0.043	25	NC_040276.1 (26685611..26701504)	*LOC114113403*	− 1.269	0.043	2	NC_040253.1 (254373219..254373344)
*LOC114111267*	− 2.211	0.043	X	NC_040278.1 (124425407..124431657 complement)	*LOC114114238*	− 1.687	0.043	3	NC_040254.1 (37341860..37341944)
*LOC114114829*	− 2.488	0.044	5	NC_040256.1 (13716402..13733880 complement)	*LOC114114239*	− 1.687	0.043	3	NC_040254.1 (37473153..37473237)
*LOC105604300*	− 2.148	0.046	22	NC_040273.1 (37630338..37678516 complement)	*LOC114109206*	− 0.809	0.043	18	NC_040269.1 (66379574..66379648)
					*LOC114114258*	0.775	0.044	3	NC_040254.1 (2846369..2846500)
					*LOC114117079*	− 1.328	0.044	11	NC_040262.1 (42450874..42450944)
					*LOC114118597*	− 1.155	0.044	15	NC_040266.1 (36567285..36567368 complement)
					*LOC114109217*	− 0.838	0.044	18	NC_040269.1 (66412057..66412132)
					*LOC114110527*	− 1.229	0.044	23	NC_040274.1 (41868170..41868239 complement)
					*LOC114111112*	− 1.184	0.044	26	NC_040277.1 (20663121..20663192)
					*LOC114111313*	− 0.944	0.045	1	NC_040252.1 (110698595..110698731)
					*LOC114111312*	− 1.532	0.045	1	NC_040252.1 (110703684..110703818)
					*LOC114118119*	− 1.187	0.045	14	NC_040265.1 (20003806..20003875)
					*LOC114110217*	− 1.257	0.045	21	NC_040272.1 (745578..745649 complement)
					*LOC114111827*	− 1.21	0.045	X	NC_040278.1 (66181207..66181276)
					*LOC114117658*	− 1.26	0.046	13	NC_040264.1 (84551659..84551730)

ncRNA genes in the fetal LV tissue of the T treated male fetuses enriched at adjusted-p value (padj) < 0.05 and absolute log2 fold change (log2FC) > 0.5 are represented. No ncRNA were differentially expressed in the female fetal LV tissue in response to T treatment.

#### snoRNA

Analysis of snoRNA modulated by prenatal T treatment revealed that out of 27 differentially expressed one snoRNA *LOC114114258 (*small nucleolar RNA, SNORA17*)* was upregulated while demonstrating a general trend of downregulation of 26 different snoRNA (Fig. 7, Table 7 listed with their chromosomal locations and Supplemental Table S11).

### ncRNA-mRNA pairs correlated in T treated male

**Figure 8:**
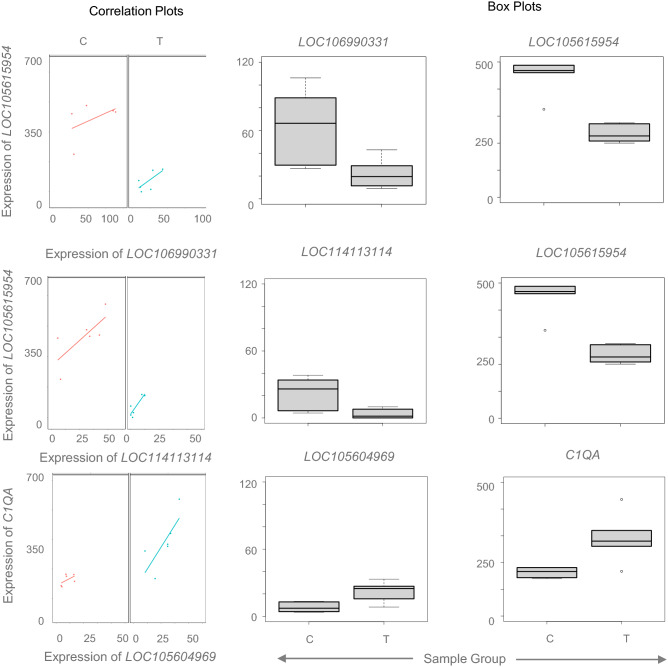
Correlation plots and average expression values for coding and lncRNA in Prenatal T-treated males. Box plots representing the average normalized counts for lncRNA and mRNA pair that showed significant correlation are shown in the right two columns. The treatment groups control (C) or prenatal T (T) are denoted on the X-axis and the average normalized counts on the Y-axis. On the left is the representation for the correlation between the corresponding lncRNA-mRNA pair represented in orange lines for the controls and in green lines for prenatal T-treated animals.

Because of absence of significantly differentially expressed ncRNA in the T-treated female fetuses, no ncRNA-mRNA correlation was performed. In contrast, in the male, lncRNA and snoRNA showed significant differential expression in response to exposure to excess T. Correlation analysis using DGCA revealed that 3 lncRNA-mRNA pairs were significantly correlated, while similar correlation could not be identified with snoRNA-mRNA pairs. Out of the three lncRNA-mRNA pairs, 2 lncRNAs, *LOC106990331* and *LOC114113114* correlated with the uncharacterized gene *LOC105615954* while lncRNA *LOC105604969* correlated with the gene *C1QA* (Fig. 8, Supplemental Table S12). For all the three lncRNA-mRNA pairs, the corresponding lncRNA and mRNA were directionally concordant and thereby showing positive correlation.

## Discussion

The findings from this study demonstrated that gestational T treatment induced sex-specific dysregulation in expression of coding RNA and non-coding RNA in the fetal LV tissue at day 90 gestational life. Furthermore, prenatal T excess led to sexual dimorphic alteration in gene network pathways associated with metabolism, response to stress and cell cycle activity. Gene network pathways associated with mitochondrial structure and function, histone modification and DNA methylation were dysregulated only in gestational T treated fetal female LV and those involved with immune response, angiogenesis and vascular function were dysregulated only in fetal males LV. Relevance of such findings and their relation to cardiovascular system function and structure will be discussed below.

### Sex differences in cardiac coding and non-coding RNAs

There are known sex differences in coding transcriptome in the heart. For example, genes on sex chromosomes are differentially expressed in male and female hearts in rodents and humans^38^. Furthermore, certain genes on autosomal chromosomes such as natriuretic peptides like *Nppb* also have sex specific expression^38–40^. In our study we note genes expressed on sex chromosome (X chromosome) including *EIF2S3 and DIPK2B* (on the female X chromosome)*,* and *SHROOM2* and *GPR143* (on the male X chromosome) to be differentially regulated between males and females, concurring with previous studies^38,41^.

Although, a previous study has assessed fetal ovine heart transcriptome at early and late gestation^42^, to our knowledge data is lacking on sexual dimorphism in cardiac transcriptome in early fetal life in sheep. Consistent with findings of Locatelli et al.^42^ we also noted differential expression of gene sets associated with cellular metabolism and processes^42^. For instance, *EIF2S3* gene located on X chromosomes and *TP53* gene differentially expressed in female LV are involved in cell cycle activity^43,44^. *SHROOM 2*, a gene differentially expressed in male LV in the current study, is noted to be involved in tissue morphogenesis including vascular development during fetal life^45^. Similarly, *TAF1A*, a gene involved in regulation of cardiac morphology^46^ was differentially expressed in male LV whereas *KDM6A*, also involved in regulation of cardiac structure^47^ was differentially expressed in female LV. Additionally, we also noted total number of differentially regulated genes were higher in females then males, an observation also reported in a recent study in adult human myocardium^41^. The relevance of these sexually dimorphic molecular pathways in left ventricular hypertrophy remains to be ascertained.

In contrast to coding transcriptome, sexual dimorphism in non-coding RNA was restricted to only one differentially expressed long coding RNA *XIST* (regulates X-chromosome inactivation) that was upregulated in female LV in our model. It is known that during early embryonic development one of the X chromosomes in female is inactivated through epigenetic process involving lncRNA *XIST*^48^. Implication of this differential expression in noncoding RNA during fetal cardiac development and function is not established. However, data is emerging on the role of inactivation/activation of X chromosome in sex differences in CVD^49^. Furthermore, activation of *XIST*’s and its role in cardiac hypertrophy and fibrosis has also been reported^50^.

### Sex-specific effects of prenatal T excess on cardiac coding and non-coding RNAs

In our current study we note that prenatal T excess highly modulated pathways associated with metabolism, response to stimuli and cell cycle activity. Furthermore, the regulation of these pathways by prenatal T excess in fetal LV were sexually dimorphic. Although the abovementioned pathways are prominent in LV during early fetal life in ovine model compared to adulthood^42^ to our knowledge sexual dimorphism in the pathways mentioned above in early gestation cardiac tissue by prenatal T excess has not been reported. Our current study establishes a baseline molecular map in the LV impacted by prenatal T excess at gestational day 90 in our model in both sexes.

We also note sex specific modulation in pathways associated with mitochondrial function in day 90 fetal LV only in females with prenatal T excess. Sex specific metabolic reprogramming has been noted in a rodent model of IUGR with late gestation female fetuses manifesting upregulation in genes associated with mitochondrial function^51^. Although low birth weight is also a characteristic of prenatal T-treated female sheep^52^, in contrast to the aforementioned study, pathways associated with mitochondrial function were downregulated. The extent to which downregulation of pathways associated with mitochondrial function in early fetal life could signify adverse vs compensatory effect in programming cardiac function remains to be determined.

Furthermore, prenatal T excess led to sex specific upregulation in genes involved in immune regulation and inflammatory pathway in male fetuses in our study. Data is emerging on the role of activated inflammatory pathway in cardiac hypertrophy^53^. In this regards, modulation of pathways associated with immune regulation and inflammation, as seen in male fetuses in our study could be detrimental to the developing heart. Moreover, we do note an increase in Complement *C1Q A* chain (*C1QA*) gene, reported to be associated with cardiac hypertrophy^54^ in the male left ventricle that could be suggestive of adverse LV programming in the male LV as well. In summary, multiple pathways were impacted by prenatal T excess (Figs. 4, 5 and 6 and supplemental Tables S6 and S7). These pathways have a potential to interact with each other in modulating an adverse cardiac structural and functional outcome. To what extent the relative contributions of the pathways perturbed by T excess and how their integration with each other programs the cardiac dysfunction in later life ultimately remains to be determined.

Similar to coding transcriptome, we did note sexual dimorphism in various non-coding RNA in our ovine model with T treatment. In males T treatment modulated long coding (lncRNA) and small nucleolar RNAs (snoRNAs). Of note, lncRNA have been noted to influence epigenetic mechanism by modulating histones and DNA methylation^55^. Hence, it could be speculated that in LV of males prenatal T excess may modulate epigenetic processes via its impact on various ncRNA. Interestingly the lncRNA ribosomal protein S28 (*RPS28*), downregulated in fetal male LV with prenatal T is also one of the genes reported to be differentially regulated in end stage dilated cardiomyopathy in human LV^56^. Furthermore, modulation in snoRNA, known to guide modulation of nucleotides in ribosomal RNA^57^ has been reported to led to morphological defects during embryogenesis in studies^58,59^. Therefore, whether snoRNA modulated by T excess in the fetal male LV has an impact on LV morphogenesis remains to be determined.

### Potential cardiac biomarkers

In our current study we utilized complementary analytical approaches namely differential expression analysis, traditionally used and dimension reduction multivariate modeling (PCA and PLS-DA) analysis. Such complementary approaches have been recently used in our prenatal T excess model assessing coding and non-coding transcriptome alteration in the liver and muscle^60^. Multivariate dimensionality reduction modeling condense related information into fewer weighted variables reducing model complexity and retaining prediction power compared to univariate analysis. These techniques are useful in analyzing coding and non-coding RNA data simultaneously as many of the ncRNA are expressed in very low levels and with high number of zero values in the data which may result in reduced power to detect significance in the traditional methods using negative binomial distribution (e.g. differential expression analysis)^60^. Higher Variable Importance in Projection (VIP) value as listed in the tables indicate higher importance to the model and variables responsible for the separation of the two groups in the PLS DA model, some of these potential signatures also met the FDR adjusted significance criteria as indicated in the “Results” section. Multivariate modeling approach in our current study identified important potential cardiac biomarkers that were modulated in the left ventricle with prenatal T excess. In female fetus *LOC101118736,* a mitochondrial gene for Ovis aries *HIGD1A* (Hypoxia inducible domain family member 1A) was upregulated with prenatal T excess. *HIGD1A* protein is important for cell survival under hypoxic conditions^61^ and shown to be upregulated in various pathological stress models including hypoxia, metabolic stress, and myocardial infarction^62^. As such, it is conceivable that upregulation of *HIGD1A* gene with prenatal T excess in female fetal LV could underlie the pathological cardiac programming.

In the males prenatal T excess led to downregulation of Zygote arrest 1 (*ZAR1*), gene involved in cell cycle activity^63^. interestingly, this gene is differentially expressed in a zebrafish model of cardiac hyperplasia^64^. Downregulation of this gene in the male LV at Day 90 may be a compensatory response to prevent cardiac hyperplasia as is noted in the female fetuses at day 90^29^. Similarly, *CAPN6* gene that was also downregulated in the male LV in our study is downregulated in hearts of hypertensive rodents^65^. Downregulation of *CAPN6* in male LV could be secondary to cardiovascular dysfunction in early fetal life programmed by prenatal T excess, an aspect yet to be investigated.

### Relation between coding and non-coding RNA

ncRNA regulates coding RNA expression subsequently contributing to various biological processes. As discussed above prenatal T excess differentially modulated lncRNA and snoRNA only in males. However, there was no correlation between differentially regulated snoRNA and coding RNA in the males. However, three differentially expressed lncRNA significantly correlated with coding RNA. Two of the lncRNAs, *LOC106990331* and *LOC114113114* correlated with the uncharacterized gene *LOC105615954* also predicted as Ret Finger Protein Like B (*RFPL4B*)*.* While there is limited information of the role of *RFPL4B* It has been shown to be downregulated during myoblast differentiation in skeletal muscle^66^. Whether downregulation of *RFPL4B* belonging to Ret Finger Protein plays a role in cardiac muscle differentiation remains to be determined.

Similarly, lncRNA *LOC105604969* correlated with the gene *C1QA* in the male fetuses, a gene that was upregulated with prenatal T excess in male fetal LV. C1Q belongs to the complement system and important for both acquired and innate immune system^67^. C1Q deficiency is known to impair clearance of apoptotic cells^68,69^. C1Q also plays a role in hypertensive arterial remodeling by activating the beta-catenin signaling^70^. The role of upregulation of *C1QA* gene in LV tissue of male fetuses exposed to prenatal T excess remains to be determined but again could be postulated that this may be in response to systemic inflammation and/or vascular dysfunction.

### Translational relevance

Prenatal T-treated sheep have served as excellent model systems to understand adverse programming of multiple metabolic and reproductive organ systems over the years^71–74^. Most organ system differentiation occurs prior to birth in sheep, like human fetuses^75^. Moreover, cardiomyocyte differentiation during fetal development in sheep has similarities to humans^76–78^. Considering prenatal T excess leads to IUGR^52^ a risk factors for cardiovascular dysfunction^5–12,79^, our findings in the present study have translational relevance relative to the developmental origins of CVD in offspring.

More recently, inclusion of sex/gender as a biological variable in cardiovascular research has become an essential requirement^80^. In this regard, determining the early transcriptomic alteration in the LV of both males and females from prenatal T excess allow for identifying potential pathways and epigenetic mechanism underlying early cardiovascular perturbation seen with prenatal T excess.

In conclusion, findings of the present study demonstrate sex-specific effects of gestational T excess between days 30 and 90 of gestation on the cardiac coding and non-coding transcriptome, thereby furthering our understanding of the molecular underpinning of prenatal T excess on the heart.

### Strengths and limitations

A major strength of this study is the utilization of an animal model with strong translational relevance to both human pregnancy and cardiovascular system. Like humans, sheep are precocial species and most of the organ system differentiation occurs prior to birth^75^. Prenatal T-treated sheep have served as excellent model systems to understand gestational adverse programming of multiple metabolic organ systems^71–74^. Importantly, sheep have emerged as suitable widely used model for cardiovascular studies due to the similarity in the cardiovascular system between human and sheep^81,82^. Of relevance to this study, cardiomyocyte differentiation during fetal development in sheep is also similar to humans^76,78^. Another strength of this study is demonstration of the sex specific molecular network within the left ventricle during early fetal life.

One limitation of our study is that RNA seq were performed on cardiac left ventricle tissue. Apart from cardiomyocyte, left ventricle has other cellular components including endothelial cells^83^. Future studies should capitalize on single cell RNA sequencing and spatial genomics to further characterize the molecular underpinning of the impact of prenatal T excess on cardiomyocyte differentiation. While transcriptome analysis by RNAseq method is a robust technique compared to other techniques as reported by earlier studies^84–86^ further investigation of protein concentration and activity that are correlated with the differentially expressed mRNAs are required despite the evidence for significant correlation between coding RNA and their target protein^87^.

## Conclusions

Findings from our current study furthers our understanding on the sexually dimorphic adverse programming of the cardiac left ventricle in early fetal life. Previously we had reported some key molecular pathways impacted by prenatal T excess at day 90 fetal life and associated perturbed cardiac morphology^29^. The current study demonstrates the extensive molecular underpinnings of adverse sex-specific programming programmed by prenatal T excess that include modulation of genes regulating inflammation, cell cycle activity and mitochondrial dysfunction. These findings extend our understanding of the molecular and morphological perturbations seen in the left ventricle during day 90 of life in a translationally relevant model of hyperandrogenic disorders.

## Materials and methods

### Animals

All animal studies were conducted under approved protocols of Institutional Animal Care and Use Committee of the University of Michigan. All methods were performed in accordance with the National Research Council’s Guide for the Care and Use of Laboratory Animals. The study was carried out in compliance with ARRIVE guidelines. Experiments in this study used multiparous female Suffolk sheep, housed at the University of Michigan Sheep Research Facility (Ann Arbor, MI) and met the National Research Council’s recommendations. Animals from both control and gestational T-treated groups were co-inhabited under similar conditions, fed a similar maintenance diet to prevent obesity as described before^52^.

### Gestational T treatment and tissue harvest

Between gestational days 30–90, 100 mg T propionate (~ 1.2 mg/kg; Millipore Sigma, St. Louis, MO) suspended in corn oil was administered intramuscularly twice a week to the T treatment group. Control animals did not receive any vehicle treatment, since our prior studies demonstrated no effects of corn oil in sheep^88^. At 91.9 ± 0.2 days of gestation, ewes were sedated with 20–30 ml of pentobarbital i.v. (Nembutol Na solution, 50 mg/ml; Abbott Laboratories, Chicago, IL), and anesthesia maintained with 1–2% halothane (Halocarbon Laboratories, River Edge, NJ). The gravid uterus was exposed through a midline incision, the fetuses were removed, and the hearts harvested. The dam was the experimental unit with only one randomly selected male or female fetus used from each dam if there were more than one fetus. Fetal body and heart weights were recorded. LV tissues were separated, snap-frozen, and stored at –80 °C until utilized for RNA extraction.

### Total RNA/small RNA extraction

Fetal cardiac LV was first homogenized in Trizol (Life Technologies, Carlsbad, CA). Chloroform was added to the homogenized tissue in trizol and spun at 16000 g for 15 min to separate the organic phase from the aqueous phase. The aqueous phase was carefully removed from the organic phase, and RNA was precipitated by addition of 70% ethanol. The precipitate was spun to pellet the RNA, washed twice in 70% ethanol, and resuspended in water. RNEasy columns (Qiagen, Germantown.MD) were used as per manufacturer’s instructions, to remove genomic DNA from the resuspended RNA. The quality and integrity of purified RNA were evaluated using Agilent 2100 bioanalyzer (Agilent Technologies, Santa Clara, CA) at the University of Michigan Advanced Genomics Core. Subsequently Ribosomal RNA in the total RNA was removed using RNAse H to obtain RNA containing mostly mRNA and noncoding RNA. In the “Results” section, while discussing total RNA, the focus is only on the coding RNA since non-coding RNA (ncRNA) was also assessed separately by small RNA sequencing. For this study, 6 male and 6 female from each of control and gestational T treated groups were used for transcriptome sequencing. Fetal weights for control females and males used in this study averaged 0.65 ± 0.10 and 0.69 ± 0.07 kg, respectively. Corresponding fetal weights for T-treated females and males were 0.59 ± 0.18 and 0.63 ± 0.22 kg, respectively and did not differ from controls. The heart weights averaged 4.51 ± 0.90 g for control females, 4.73 ± 0.76 g for control males, 4.33 ± 1.50 g for T treated females and 4.30 ± 1.45 g for T treated males and there was no significant difference in heart weight between groups.

### Library construction and sequencing

The coding RNA libraries for high-throughput sequencing were generated using NEBNext RNA Ultra II reagents (Ipswich, Massachusetts) and the generated libraries were paired-end sequenced on the NovaSeq platform at 150 cycles. ncRNA libraries were prepared from total RNA using NEBNext smallRNA kit (New England BioLabs, Ipswich, MA). The libraries quality control metrics were determined using agilent bioanalyzer and the generated libraries were sequenced on the NovaSeq platform. The integrity of the generated libraries for both the coding or noncoding libraries were determined using Agilent 2100 bioanalyzer.

### Coding RNA trimming

Raw fastq files were trimmed to remove adapter sequences and low-quality reads using trimmomatic. The parameters used for trimmomatic included maximum mismatch count, palindrome clip, and clip threshold score parameters for removing Illumina adapters: 2:30:10. Further all leading and trailing bases with quality threshold below 3 and reads that dropped below a quality score of 15 while scanning bases with a 4 base pair window were also removed.

### smallRNA trimming

Raw reads from smallRNA were trimmed using cutadapt (v3.2) and specifically trimmed from the 5’ end using the sequence ‘AGATCGGAAGAGCACACGTCTGAACTCCAGTCAC’ and reads that were less than 17 bp and low-quality reads that did not match the default quality control scores acceptable for cutadapt were removed.

### Quality control metrics

Quality control metrics for both raw and trimmed files were evaluated using fastqc and summarized using multiqc.

### Alignment

Trimmed reads were aligned to sheep reference genome (Oar_rambouillet_v1.0) using Spliced Transcripts Alignment to a Reference (STAR) aligner (v2.6.0c). Feature Counts (v1.6.1) was used to count aligned fragments and then differential expression performed.

### Differential gene expression testing

DESEq2(1.24.0) utilizing negative binomial distribution on counts was used for determining the differential expression of both coding and ncRNA. Sex and sex-specific treatment effects in coding and ncRNA expression in fetal cardiac tissue were determined by comparing (1) control male with control female, (2) gestational T-treated male vs. control male and (3) gestational T-treated female vs. control female. For both coding and ncRNA differentially expressed transcripts that met the False Discovery Rate < 0.05 and absolute log2Fold Change > 0.5 was considered significant. Enhanced Volcano package was used to generate volcano plots for visualization of differentially expressed transcripts. Heatmaps for representing the normalized expression counts for each animal was generated using heatmap.2 package. Both the volcano plots and heatmaps were generated using R statistical software (v3.5.1).

### Data reduction

Dimensionality reduction modeling was performed using SIMCA 17 (Sartorius Stedim Data Analytics AB, Sweden). Normalized counts for coding and ncRNA from male and female LV were imported to SIMCA software for the analysis. Multivariate modeling was performed using unit variance (UV) scaling (mean centered and divided by the standard deviation). To get an overview of the data and identify possible outliers and patterns/groupings in the data, unsupervised principal component analysis (PCA) was performed and two-dimensional and three dimensional (3D) PCA plots were explored. A tolerance ellipse based on Hotelling’s T2 (multivariate generalization of Student’s t-distribution) was used to identify strong outliers outside the ellipses. Identified outliers were removed and PCA was performed to investigate patterns in the data (one outlier was removed from lncRNA, snoRNA and snRNA from female cardiac tissue). Partial Least Square Discriminant Analysis (PLS-DA) was performed to investigate differences in the male/female coding and non-coding transcriptome profiles. PLS-DA, a supervised analysis was performed, where the transcriptome profiles (coding / ncRNA) were used as X variables and sex / treatment were used as an outcome Y variable to generate models. The scatter score plot of components 1 and 2 were explored to visualize differences in the two groups (classes). Observations that are close to each other have more similar transcriptome profiles compared to the observations that are distant from each other. The clustering, patterns, and groupings were observed. The Variables Importance in Projection (VIP) were computed and investigated to identify potential signatures of exposure to T excess on LV tissue. VIP value above 1 indicates importance to the model. List of top VIP values were generated as important genes responsible for separation of the groups on PLS-DA score plot. All PLS-DA models used for potential gene signature selection had good fit and predictive ability (R2 above 0.7 and Q2 above 0.5) except female miRNA, which had low Q2 value (0.27), Furthermore, significance (based on differential expression fold change) of these signatures were investigated to identify potential biomarkers of exposure to T excess in the LV of male and female fetuses.

### Gene set enrichment analysis

Human orthologs of sheep genes were determined using BioMart and enrichment of pathways using RNA-enrich^89^. Our parameters for using this tool included using a RNA-enrich tools default setting of a minimum of 10 genes and a maximum of 99,999 genes for the gene set size. Specifically, RNA-enrich uses a *p*-value cut-off free enrichment methodology and genes were tested for enrichment by comparison against 6 databases including Biocarta pathway, EHMN metabolic pathways, Gene Ontology, KEGG pathway, Panther pathway, and transcription factor databases. Pathways that met false discovery rate < 0.01 were considered significant and comparisons made between control males and females (sex effect) or between T-treated and controls for each sex (sex-specific treatment effect). Odds Ratio (OR) for all comparisons are described in this study and the findings were visualized using UpSet plots generated by Upset R. All data were processed using R statistical software (v3.5.1).

### Correlation of coding RNA and ncRNA

To determine the relationship between coding RNA and ncRNA in males, the counts for differentially expressed coding RNA genes in males were matched to ncRNA within their respective samples from the same set of animals. The correlation matrix was generated by differential gene correlation analysis (DGCA). ncRNA-coding RNA pairs with Pearson correlation coefficients with *p*-value < 0.05 (control vs. treatment) were considered significantly correlated for that group. The female fetuses did not have any significant differentially expressed ncRNA to perform correlation analysis.

Generation of chromosome locations for all the genes listed in this study: All genes as listed in the tables are accompanied by chromosomal locations. The chromosomal locations for the coding RNA or ncRNA listed in the tables were obtained from NCBI gene database. All locations correspond to those in the assembly for Oar_rambouillet_v1.0 whose accession number is GCF_002742125.1.

## Supplementary Information

Supplementary Figure S1.

Supplementary Figure S2.

Supplementary Figure S3.

Supplementary Figure S4.

Supplementary Figure S5.

Supplementary Figure S6.

Supplementary Legends.

Supplementary Table S1.

Supplementary Table S2.

Supplementary Table S3.

Supplementary Table S4.

Supplementary Table S5.

Supplementary Table S6.

Supplementary Table S7.

Supplementary Table S8.

Supplementary Table S9.

Supplementary Table S10.

Supplementary Table S11.

Supplementary Table S12.

## Data Availability

All data generated or analyzed during this study are included in this published article and its supplementary information files.
