# Supplementary figures and images for: Developmental programming: adverse sexually dimorphic transcriptional programming of gestational testosterone excess in cardiac left ventricle of fetal sheep
Source: Sci Rep. 2023 Feb 15;13:2682. doi: 10.1038/s41598-023-29212-9 (PMC9932081; doi:10.1038/s41598-023-29212-9)

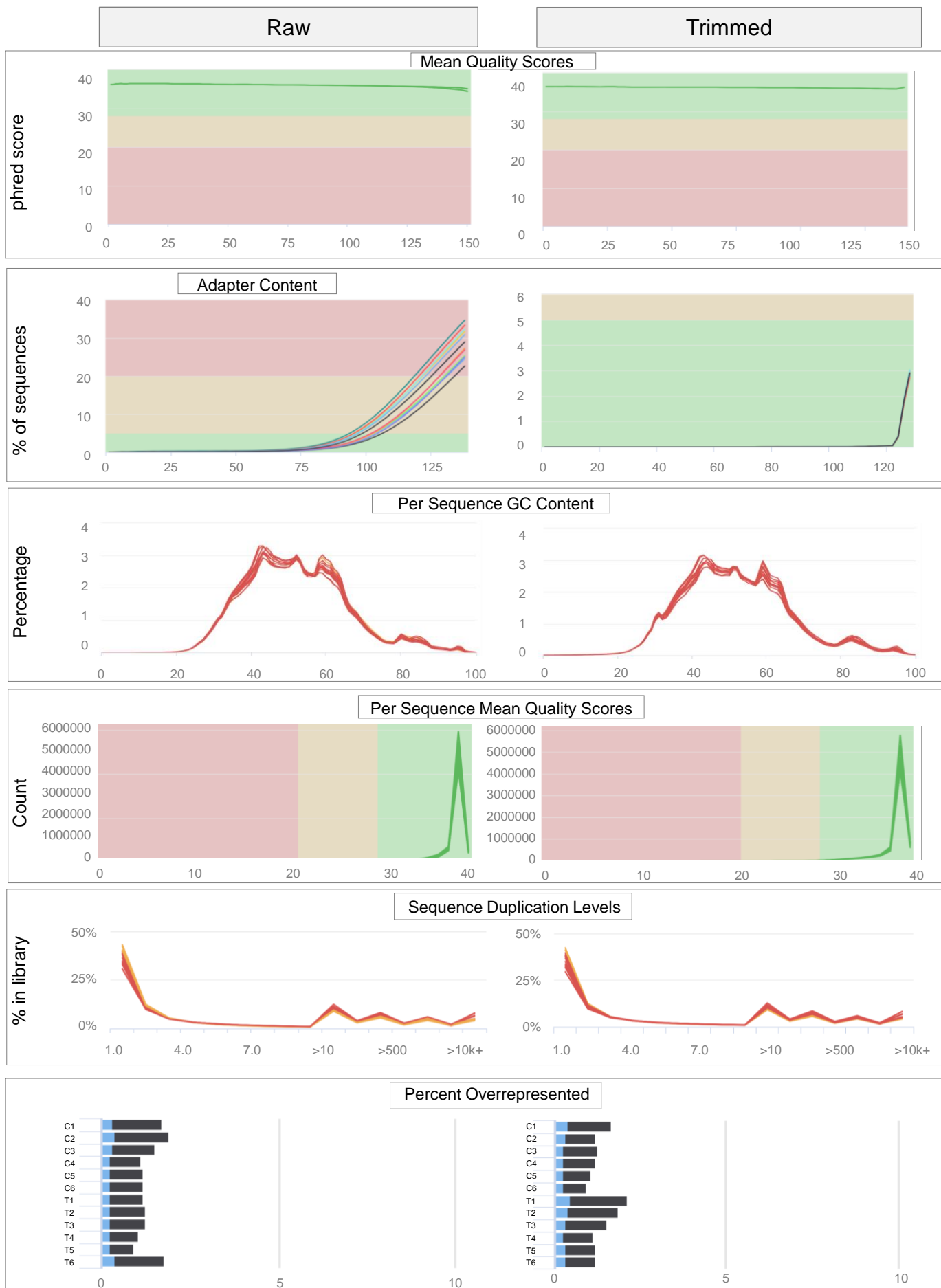

Figure S1

Supplement: Supplementary file 1 — Supplementary Figure S1. [file 41598_2023_29212_MOESM1_ESM.pdf]

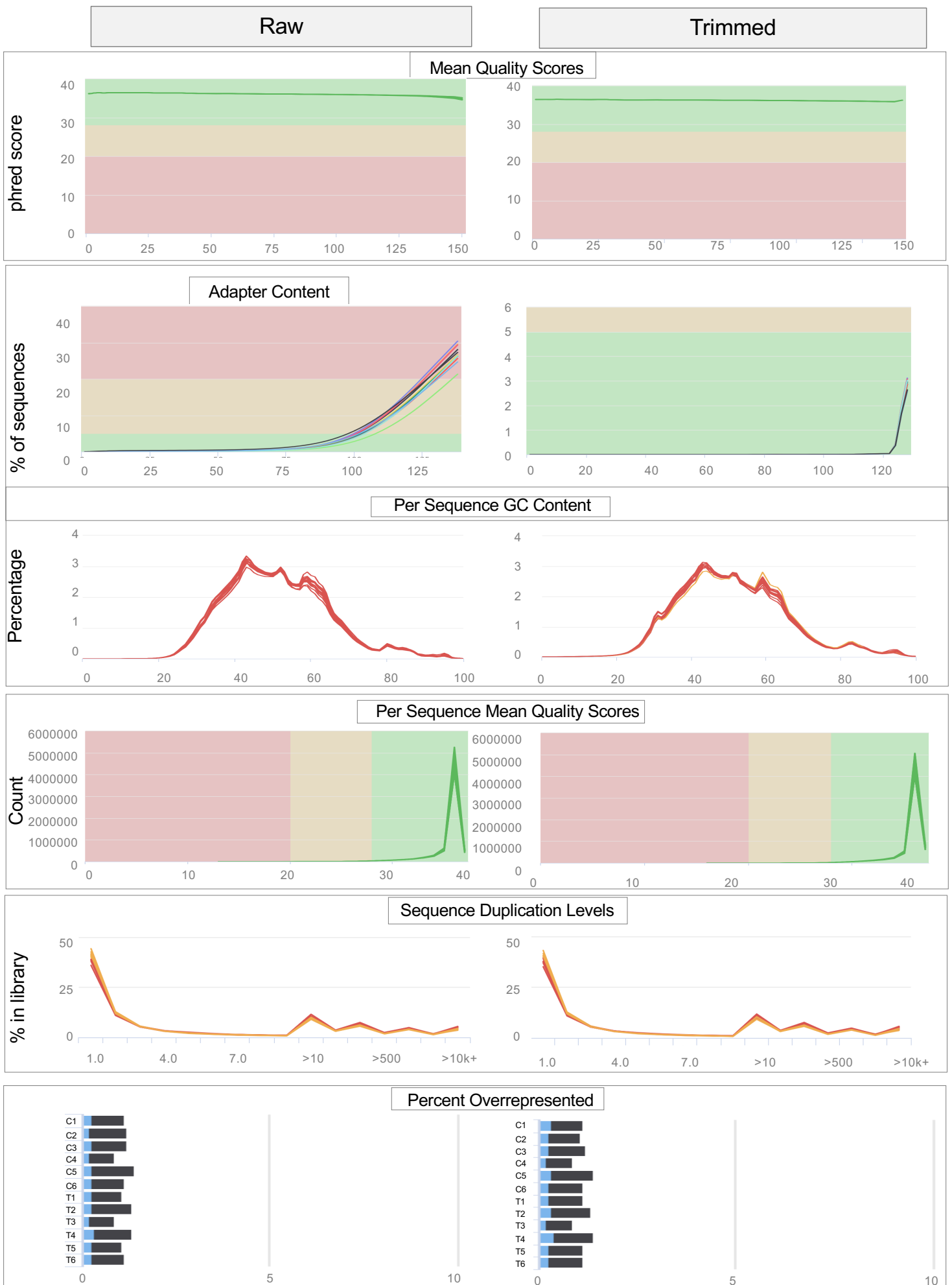

Figure S2

Supplement: Supplementary file 2 — Supplementary Figure S2. [file 41598_2023_29212_MOESM2_ESM.pdf]

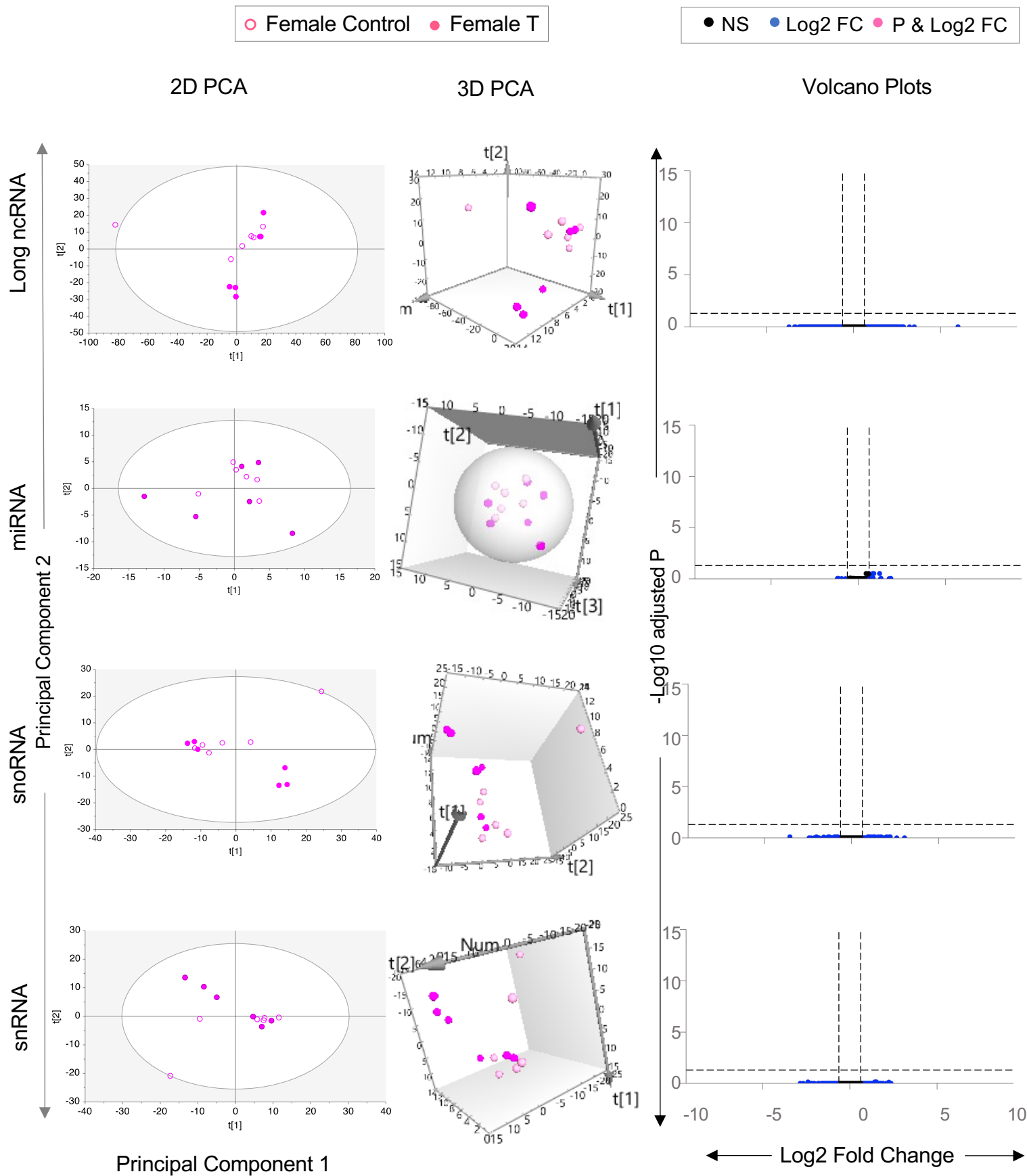

Figure S3 Female ncRNA including the outlier

Supplement: Supplementary file 3 — Supplementary Figure S3. [file 41598_2023_29212_MOESM3_ESM.pdf]

○ Male Control ● Male T

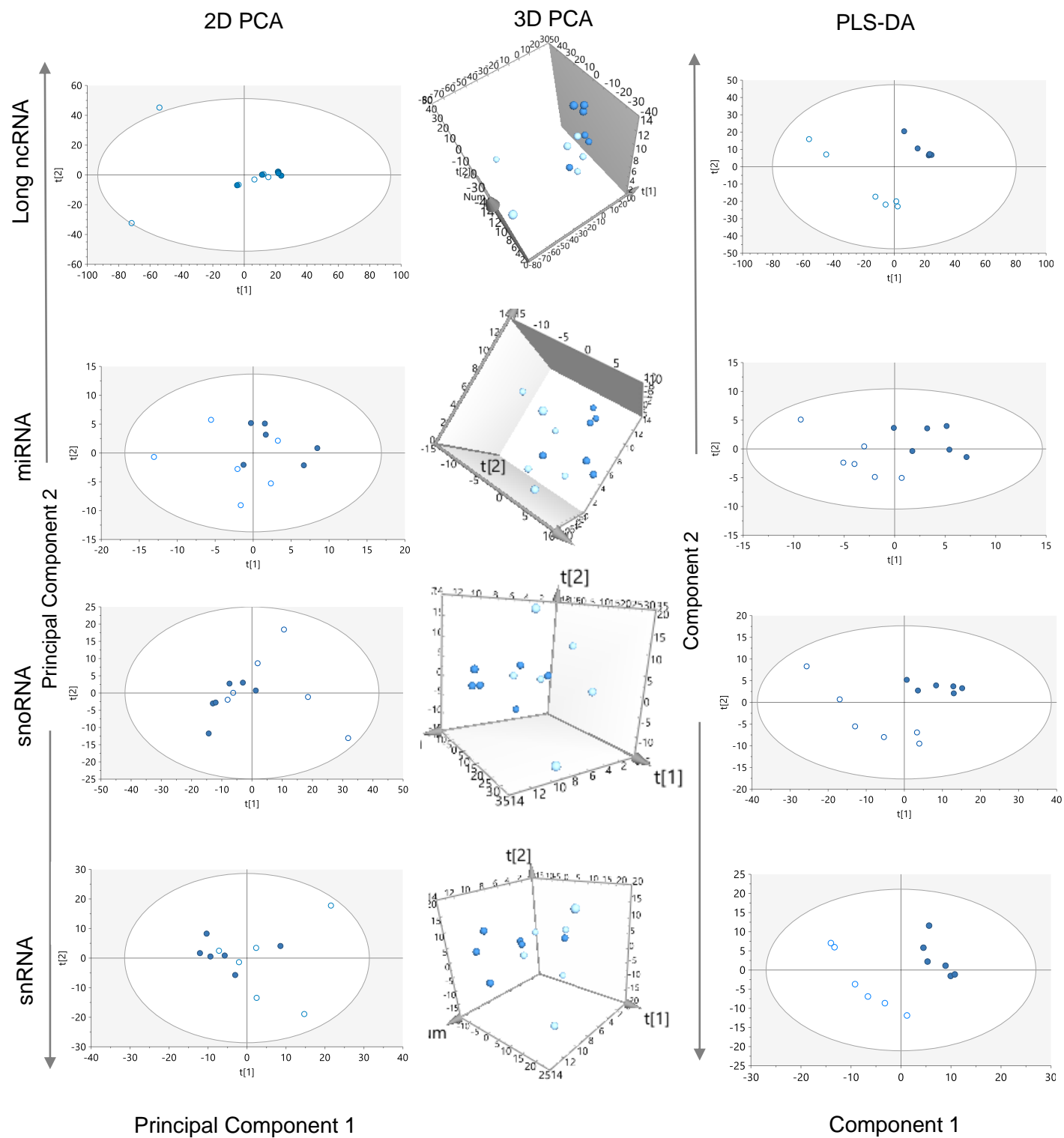

Figure S5 Male ncRNA PCA and PLS DA

Supplement: Supplementary file 5 — Supplementary Figure S5. [file 41598_2023_29212_MOESM5_ESM.pdf]

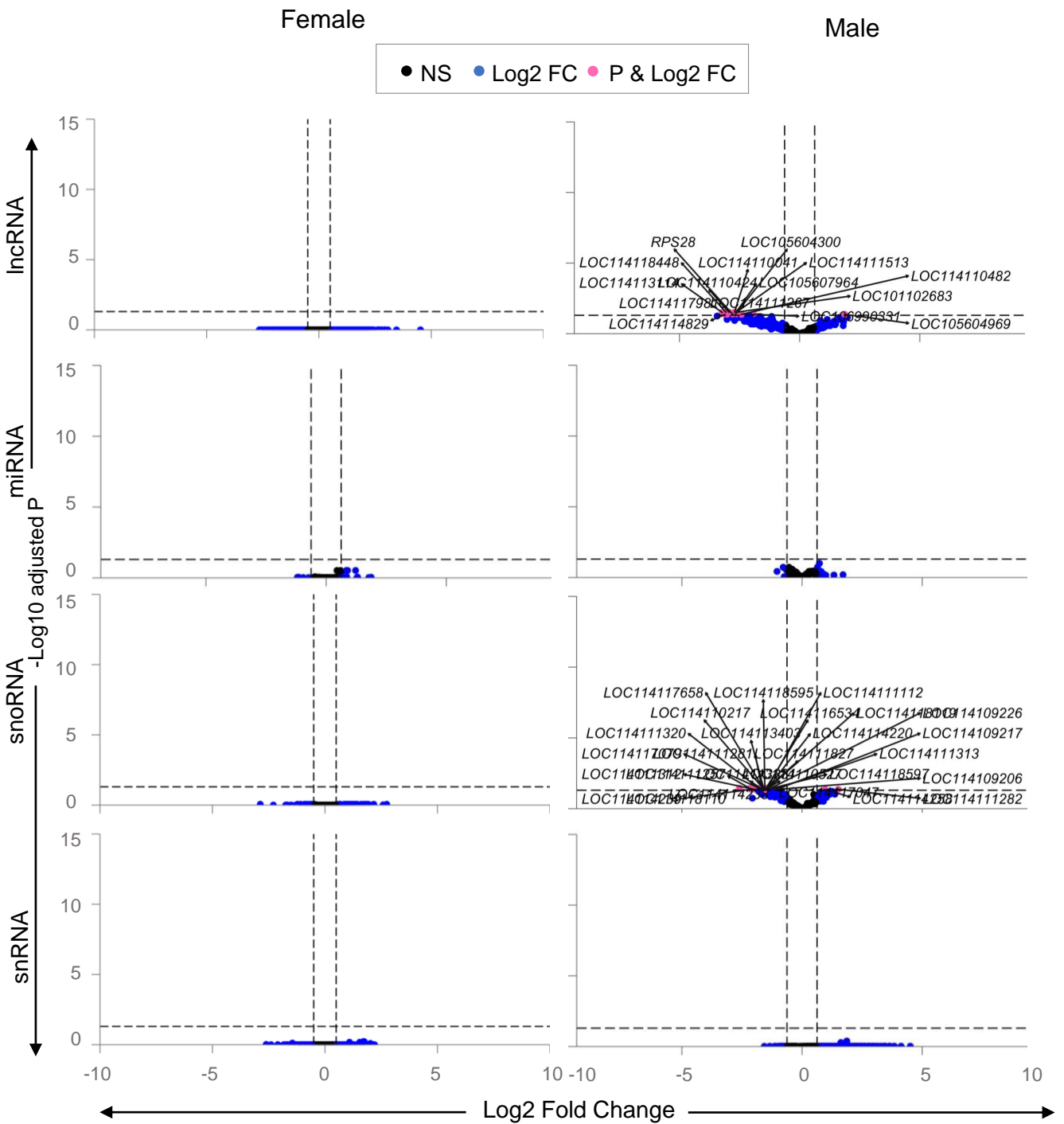

Fig. S6. Volcano plots ncRNA Female and Male Control vs Prenatal T

Supplement: Supplementary file 6 — Supplementary Figure S6. [file 41598_2023_29212_MOESM6_ESM.pdf]
