# Supplementary material for: Developmental programming: adverse sexually dimorphic transcriptional programming of gestational testosterone excess in cardiac left ventricle of fetal sheep
Source: Sci Rep. 2023 Feb 15;13:2682. doi: 10.1038/s41598-023-29212-9 (PMC9932081; doi:10.1038/s41598-023-29212-9)
Supplement: Supplementary file 4 — Supplementary Figure S4. [file 41598_2023_29212_MOESM4_ESM.pdf]

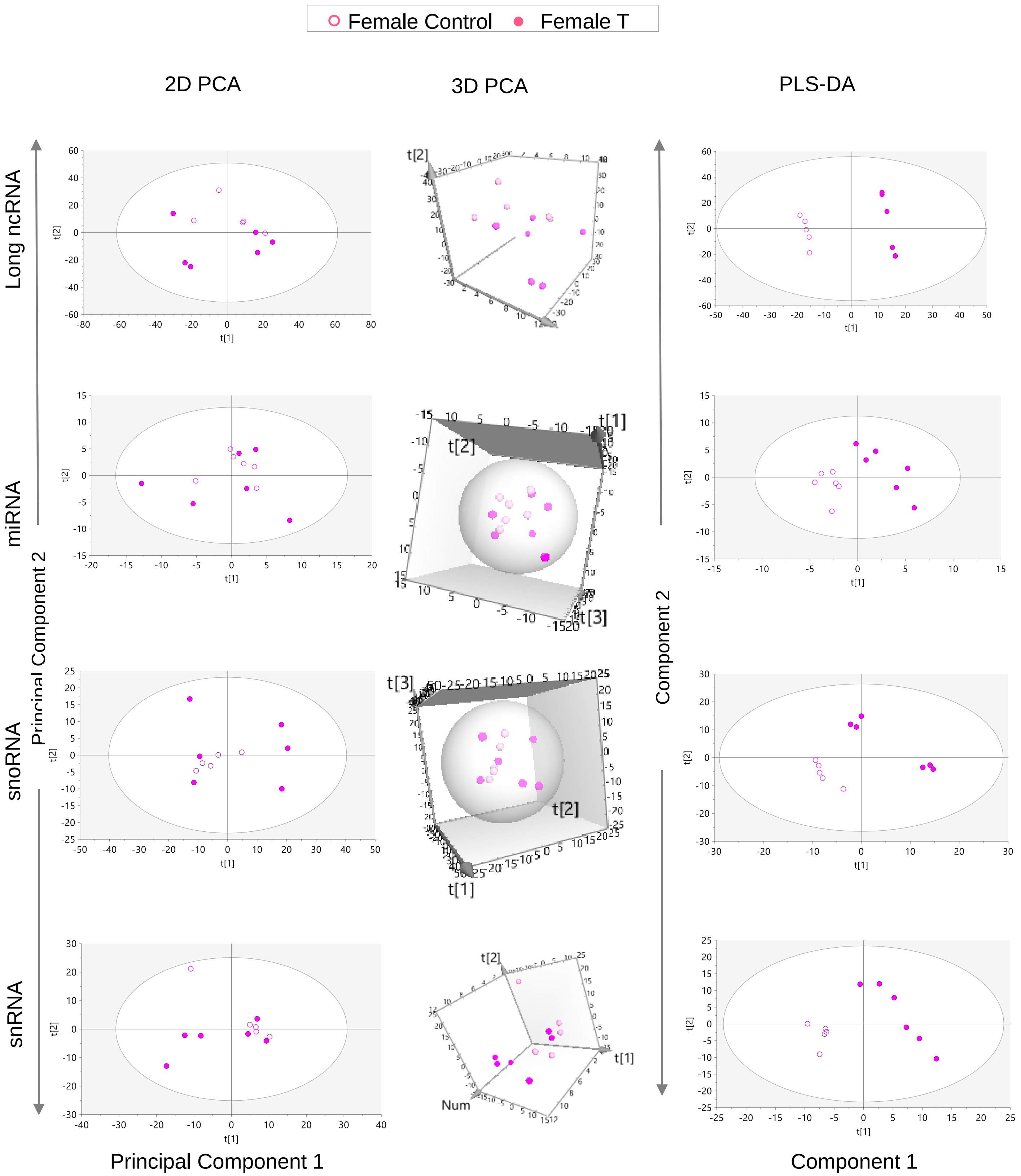

Figure S4 Female ncRNA PCA and PLS DA after removing outliers
