# Supplementary material for: Developmental programming: adverse sexually dimorphic transcriptional programming of gestational testosterone excess in cardiac left ventricle of fetal sheep
Source: Sci Rep. 2023 Feb 15;13:2682. doi: 10.1038/s41598-023-29212-9 (PMC9932081; doi:10.1038/s41598-023-29212-9)
Supplement: Supplementary file 7 — Supplementary Legends. [file 41598_2023_29212_MOESM7_ESM.docx]

Developmental Programming: Adverse sexually dimorphic transcriptional programming of gestational testosterone excess in cardiac left ventricle of fetal Sheep.

Venkateswaran Ramamoorthi Elangovan^1^*, Nadia Saadat^1^*, Adel Ghnenis^1^, Vasantha Padmanabhan^1^, Arpita K Vyas^2#^

^1^Department of Pediatrics, University of Michigan, Ann Arbor, Michigan

^2^California Northstate University, College of Human Medicine, Elk Grove, CA, USA

* These authors share first authorship of this work.

**Supplemental Figure Legends:**

**Supplemental Fig. S1.** Quality control of raw and trimmed sequencing data assessed by fastqc for female fetal cardiac tissue is depicted. The phred score, % of sequences containing adapter sequences, percent GC content, per sequence mean quality scores, sequence duplication levels after grouping samples using multiqc are shown.

**Supplemental Fig S2.** Quality control of raw and trimmed sequencing data assessed by fastqc for male fetal cardiac tissue is depicted. The phred score, % of sequences containing adapter sequences, percent GC content, per sequence mean quality scores, sequence duplication levels after grouping samples using multiqc are shown.

**s**

**Supplemental Fig S3. Clustering and PLS DA for ncRNA in Female animals treated with prenatal T (Outlier included).** The 2D, 3D PCA plots and PLS-DA showing clustering between female control (pink open circle) and prenatal T-treated fetal cardiac tissue (pink closed circle) groups after removing the outlier samples in the ncRNA (lncRNA, snoRNA, snRNA) from fetal cardiac tissue are shown. For the PCA the 2D plots, 3D plots are plotted with principal component 1 on X-axis and principal component 2 on Y-axis, and PLS-DA plots are plotted with component 1 on X-axis and component 2 on Y-axis, with each point representing one animal.

**Supplemental Fig S4.** **Clustering and PLS-DA Plots for ncRNA in Female animals treated with prenatal T (Outlier removed).** The 2D, 3D PCA plots and PLS-DA showing clustering between female control (pink open circle) and prenatal T-treated fetal cardiac tissue (pink closed circle) groups after removing the outlier samples in the ncRNA (lncRNA, snoRNA, snRNA) from fetal cardiac tissue are shown. For the PCA the 2D plots, 3D plots are plotted with principal component 1 on X-axis and principal component 2 on Y-axis, and PLS-DA plots are plotted with component 1 on X-axis and component 2 on Y-axis, with each point representing one animal.

**Supplemental Fig S5. Clustering and PLS-DA Plots for ncRNA in Male animals treated with prenatal T.** The 2D, 3D PCA plots and PLS-DA showing clustering between control (blue open circle for male) and prenatal T-treated fetal cardiac tissue (blue closed circle for male) groups in the ncRNA from fetal cardiac tissue are shown. For the PCA the 2D plots, 3D plots are plotted with principal component 1 on X-axis and principal component 2 on Y-axis, and PLS-DA plots are plotted with component 1 in the X-axis and component 2 in the Y-axis, with each point representing one animal.

**Supplemental Fig S6. Differential expression of ncRNA in female and male prenatal T treated animals.** Volcano plot showing differential ncRNA (lncRNA, miRNA, snoRNA, snRNA) expression in the female and male cardiac tissues of prenatal T-treated animals compared against the control animals. Differential expression analysis in the female was performed after removing the outlier from lncRNA, snoRNA, snRNA. The log2 fold change values are represented against the X-axis and -log10 adjusted p-values are represented along the Y-axis. Pink dots represent those ncRNA that met absolute log2FC>0.5 and p-adjusted cut-off of <0.05. Black dots represent ncRNA genes that did not meet either p-adjusted cut-off of < 0.05 or absolute log2FC > 0.5 and blue dots represent genes which met absolute log2 fold change > 0.5 but did not meet the adjusted p-values < 0.05.
