# Supplementary material for: Developmental programming: adverse sexually dimorphic transcriptional programming of gestational testosterone excess in cardiac left ventricle of fetal sheep
Source: Sci Rep. 2023 Feb 15;13:2682. doi: 10.1038/s41598-023-29212-9 (PMC9932081; doi:10.1038/s41598-023-29212-9)
Supplement: Supplementary file 16 — Supplementary Table S9. [file 41598_2023_29212_MOESM16_ESM.docx]

**Table S9: Top 30 potential noncoding biomarkers in female fetal LV tissue exposed to excess T**

| lncRNA | | | |
| --- | --- | --- | --- |
| ID | VIP | log2FC | p-adj |
| *LOC106990172* | 3.3 | -2.315 | 0.999 |
| *LOC105603376* | 3.3 | -2.315 | 0.999 |
| *LOC105607690* | 2.95 | 1.449 | 0.999 |
| *LOC105609379* | 2.93 | 1.724 | 0.999 |
| *LOC114112959* | 2.9 | -2.7 | 0.999 |
| *LOC105603399* | 2.89 | -2.96 | 0.999 |
| *LOC114116900* | 2.87 | -2.48 | 0.999 |
| *LOC114116922* | 2.77 | 0.356 | 0.999 |
| *LOC114115600* | 2.77 | 0.688 | 0.999 |
| *LOC114117256* | 2.74 | -2.086 | 0.999 |
| *LOC105604994* | 2.74 | 0.888 | 0.999 |
| *LOC101108158* | 2.73 | 0.265 | 0.999 |
| *LOC114116373* | 2.72 | -2.259 | 0.999 |
| *LOC114109710* | 2.65 | -1.61 | 0.999 |
| *LOC106990346* | 2.61 | -1.419 | 0.999 |
| *LOC114114611* | 2.61 | -2.692 | 0.999 |
| *LOC114117229* | 2.59 | -2.449 | 0.999 |
| *LOC105609117* | 2.58 | 0.611 | 0.999 |
| *LOC105605187* | 2.49 | -2.195 | 0.999 |
| *LOC105604230* | 2.48 | -1.841 | 0.999 |
| *LOC105613564* | 2.46 | 1.18 | 0.999 |
| *LOC114112790* | 2.45 | -1.399 | 0.999 |
| *LOC114111172* | 2.43 | -1.762 | 0.999 |
| *LOC105604160* | 2.41 | -2.476 | 0.999 |
| *LOC105604676* | 2.4 | 0.626 | 0.999 |
| *LOC114114096* | 2.4 | -1.562 | 0.999 |
| *LOC105602686* | 2.4 | -1.133 | 0.999 |
| *LOC105610932* | 2.4 | -1.92 | 0.999 |
| *LOC105608783* | 2.37 | -2.027 | 0.999 |
| *LOC105609538* | 2.31 | 1.263 | 0.999 |
| miRNA | | | |
| Gene | VIP | log2FC | p-adj |
| *MIR431* | 2.09 | 0.492 | 0.437 |
| *MIR30C* | 2.02 | 0.361 | 0.301 |
| *MIR539* | 1.87 | 1.046 | 0.884 |
| *MIR376B* | 1.84 | -0.588 | 0.956 |
| *MIR495* | 1.8 | 0.48 | 0.301 |
| *MIR323C* | 1.79 | 0.673 | 0.301 |
| *MIR380* | 1.74 | 0.712 | 0.301 |
| *MIR99A* | 1.63 | -0.373 | 0.832 |
| *MIR152* | 1.59 | -0.227 | 0.884 |
| *MIR22* | 1.57 | -0.361 | 0.832 |
| *MIR218A* | 1.54 | -0.356 | 0.832 |
| *MIR3955* | 1.52 | 0.98 | 0.301 |
| *MIR200C* | 1.51 | -0.91 | 0.906 |
| *MIR199A* | 1.5 | -0.235 | 0.906 |
| *MIR376A* | 1.4 | 0.624 | 0.906 |
| *MIR668* | 1.37 | 0.354 | 0.957 |
| *MIR23B* | 1.36 | -0.344 | 0.884 |
| *MIR23A* | 1.34 | -0.405 | 0.884 |
| *MIR103* | 1.3 | -0.115 | 0.957 |
| *MIR154A* | 1.25 | 0.683 | 0.832 |
| *MIR381* | 1.25 | 0.356 | 0.906 |
| *MIR362* | 1.24 | 0.437 | 0.884 |
| *MIR30B* | 1.21 | 0.546 | 0.832 |
| *MIR107* | 1.19 | -0.088 | 0.957 |
| *MIR181A-1* | 1.17 | -0.273 | 0.918 |
| *MIR433* | 1.13 | 0.293 | 0.906 |
| *MIR181A-2* | 1.12 | -0.248 | 0.956 |
| *MIR194* | 1.09 | -0.157 | 0.957 |
| *MIR26A* | 1.07 | -0.148 | 0.957 |
| *MIR376D* | 1.07 | 1.496 | 0.906 |
| snoRNA | | | |
| ID | VIP | log2FC | p-adj |
| *LOC114116537* | 2.38 | 1.212 | 1 |
| *LOC114111811* | 2.06 | -2.825 | 0.796 |
| *LOC114116768* | 2.05 | -0.549 | 1 |
| *LOC114115071* | 2.04 | -1.018 | 0.796 |
| *LOC114110373* | 2.01 | 1.333 | 0.851 |
| *LOC114116540* | 1.97 | 1.133 | 0.959 |
| *LOC114118586* | 1.94 | 0.321 | 1 |
| *LOC114118127* | 1.91 | -0.518 | 1 |
| *LOC114118123* | 1.83 | -0.828 | 0.993 |
| *LOC114111355* | 1.81 | 1.28 | 1 |
| *LOC114109492* | 1.8 | -0.641 | 0.993 |
| *LOC114115938* | 1.79 | 0.793 | 1 |
| *LOC114116757* | 1.78 | -1.271 | 0.836 |
| *LOC114109762* | 1.77 | -0.784 | 0.809 |
| *LOC114117066* | 1.74 | 1.237 | 0.796 |
| *LOC114110523* | 1.74 | -1.86 | 1 |
| *LOC114109227* | 1.74 | 0.027 | 1 |
| *LOC114117380* | 1.73 | 0.135 | 1 |
| *LOC114117677* | 1.72 | 2.437 | 0.993 |
| *LOC114117653* | 1.71 | -0.625 | 1 |
| *LOC114113370* | 1.7 | -1.621 | 0.993 |
| *LOC114115450* | 1.69 | -0.955 | 0.993 |
| *LOC114111312* | 1.69 | -0.803 | 0.876 |
| *LOC114117364* | 1.69 | -0.142 | 1 |
| *LOC114115072* | 1.67 | 0.302 | 1 |
| *LOC114110907* | 1.67 | 0.302 | 1 |
| *LOC114112106* | 1.67 | -0.723 | 1 |
| *LOC114108879* | 1.66 | -0.81 | 1 |
| *LOC114111366* | 1.66 | 1 | 1 |
| *LOC114109181* | 1.65 | 1.706 | 1 |
| snRNA | | | |
| Primary ID | VIP | log2FC | padj |
| *LOC114115102* | 3.814 | -2.270 | 0.997 |
| *LOC114117071* | 3.352 | -1.639 | 0.997 |
| *LOC114115955* | 3.206 | -1.291 | 0.997 |
| *LOC114110953* | 3.102 | -1.420 | 0.997 |
| *LOC114115956* | 2.913 | -1.207 | 0.997 |
| *LOC114114660* | 2.868 | -1.916 | 0.739 |
| *LOC114117122* | 2.812 | -0.880 | 0.997 |
| *LOC114110196* | 2.790 | 1.140 | 0.739 |
| *LOC114116739* | 2.734 | -2.133 | 0.997 |
| *LOC114114281* | 2.733 | -0.993 | 0.997 |
| *LOC114110371* | 2.706 | -0.139 | 0.997 |
| *LOC114115131* | 2.562 | 1.792 | 0.769 |
| *LOC114113426* | 2.466 | 0.323 | 0.997 |
| *LOC114117038* | 2.445 | -0.388 | 0.997 |
| *LOC114117121* | 2.413 | -0.621 | 0.997 |
| *LOC114110491* | 2.410 | 1.514 | 0.997 |
| *LOC114108896* | 2.378 | 1.352 | 0.997 |
| *LOC114115113* | 2.376 | -1.630 | 0.997 |
| *LOC114117116* | 2.369 | 1.215 | 0.739 |
| *LOC114109219* | 2.369 | -0.949 | 0.997 |
| *LOC114117114* | 2.368 | 1.218 | 0.739 |
| *LOC114117112* | 2.367 | 1.217 | 0.739 |
| *LOC114117113* | 2.367 | 1.217 | 0.739 |
| *LOC114117115* | 2.355 | 1.191 | 0.739 |
| *LOC114114198* | 2.350 | 1.679 | 0.997 |
| *LOC114117117* | 2.348 | 1.185 | 0.739 |
| *LOC114117120* | 2.347 | 1.164 | 0.739 |
| *LOC114116759* | 2.328 | 0.944 | 0.997 |
| *LOC114112135* | 2.326 | 1.241 | 0.997 |
| *LOC114110972* | 2.325 | 1.265 | 0.997 |

Top 30 noncoding RNA biomarkers for lncRNA, miRNA, snoRNA, snRNA comparing control female and T-treated female fetal cardiac tissue based on variable importance in Projection values along with the corresponding log2FC and padj values obtained from DESeq2 analysis for the same determinants are represented.
