# Supplementary material for: Developmental programming: adverse sexually dimorphic transcriptional programming of gestational testosterone excess in cardiac left ventricle of fetal sheep
Source: Sci Rep. 2023 Feb 15;13:2682. doi: 10.1038/s41598-023-29212-9 (PMC9932081; doi:10.1038/s41598-023-29212-9)
Supplement: Supplementary file 17 — Supplementary Table S10. [file 41598_2023_29212_MOESM17_ESM.docx]

**Table S10: Top 30 potential noncoding biomarkers in male fetal LV tissue**

**exposed to excess T**

| lncRNA | | | |
| --- | --- | --- | --- |
| ID | VIP | log2FC | p-adj |
| *LOC114108850* | 2.300 | -2.960 | NA |
| *LOC114118304* | 2.243 | -1.366 | NA |
| *LOC105611709* | 2.217 | -3.224 | NA |
| *LOC114114817* | 2.204 | 1.017 | NA |
| *LOC114116072* | 2.149 | -0.888 | NA |
| *LOC105602307* | 2.135 | -2.779 | NA |
| *LOC105614781* | 2.073 | 1.149 | NA |
| *LOC105607140* | 2.061 | -2.280 | NA |
| *LOC105606997* | 2.049 | -1.849 | NA |
| *LOC114109100* | 2.040 | -1.654 | NA |
| *LOC114115575* | 2.018 | -2.167 | NA |
| *LOC114112786* | 2.006 | -2.137 | NA |
| *LOC114116193* | 2.000 | -0.890 | NA |
| *LOC105613097* | 1.997 | -1.922 | NA |
| *LOC114116115* | 1.989 | -1.540 | NA |
| *LOC114109081* | 1.988 | -1.267 | 0.091 |
| *LOC105603320* | 1.987 | 0.558 | 0.336 |
| *LOC114118398* | 1.968 | -1.351 | NA |
| *LOC105616720* | 1.936 | -1.984 | NA |
| *LOC114114863* | 1.885 | -1.704 | NA |
| *LOC106991094* | 1.868 | -2.132 | NA |
| *LOC114109386* | 1.862 | -1.498 | NA |
| *LOC105603919* | 1.843 | -0.770 | 0.210 |
| *LOC114113891* | 1.836 | 1.132 | 0.188 |
| *LOC114116874* | 1.830 | -2.474 | NA |
| *LOC114111042* | 1.818 | 1.958 | NA |
| *LOC114112977* | 1.809 | -1.532 | NA |
| *LOC114113884* | 1.792 | -1.776 | NA |
| *LOC114114095* | 1.779 | -2.101 | NA |
| *LOC114117250* | 1.775 | -0.882 | NA |
| miRNA | | | |
| ID | VIP | log2FC | p-adj |
| *MIR17* | 1.583 | 0.278 | 0.357 |
| *MIR369* | 1.545 | 0.581 | 0.099 |
| *MIR3958* | 1.544 | 0.073 | 0.703 |
| *MIR134* | 1.526 | -0.331 | 0.255 |
| *MIR194* | 1.381 | 0.215 | 0.481 |
| *MIR106B* | 1.373 | -0.435 | 0.186 |
| *MIR377* | 1.345 | -0.832 | 0.369 |
| *MIRLET7I* | 1.344 | -0.251 | 0.369 |
| *MIR3959* | 1.344 | 0.513 | 0.186 |
| *MIR411B* | 1.310 | 0.424 | 0.347 |
| *MIR411A* | 1.288 | 0.372 | 0.341 |
| *MIR16B* | 1.283 | -0.403 | 0.255 |
| *MIR127* | 1.274 | -0.236 | 0.369 |
| *MIR380* | 1.273 | -0.191 | 0.416 |
| *MIR494* | 1.272 | 0.228 | 0.369 |
| *MIR3955* | 1.263 | -0.619 | 0.195 |
| *MIR381* | 1.253 | -0.220 | 0.677 |
| *MIR410* | 1.239 | 0.180 | 0.546 |
| *MIRLET7B* | 1.237 | -0.264 | 0.369 |
| *MIR150* | 1.235 | -0.627 | 0.186 |
| *MIR374A* | 1.229 | 0.389 | 0.357 |
| *MIR668* | 1.229 | 0.474 | 0.518 |
| *MIR200C* | 1.226 | -0.476 | 0.768 |
| *MIR106A* | 1.189 | 0.152 | 0.718 |
| *MIR376E* | 1.182 | 0.550 | 0.518 |
| *MIR431* | 1.175 | -0.338 | 0.381 |
| *MIR541* | 1.168 | -0.401 | 0.369 |
| *MIRLET7C* | 1.138 | -0.256 | 0.369 |
| *MIR3957* | 1.137 | -0.374 | 0.408 |
| *MIR433* | 1.126 | 0.204 | 0.505 |
| snoRNA | | | |
| ID | VIP | log2FC | p-adj |
| *LOC114117374* | 2.559 | 2.314 | NA |
| *LOC114110701* | 2.357 | 0.352 | 0.391 |
| *LOC114115090* | 2.069 | -0.537 | 0.098 |
| *LOC114118628* | 2.050 | -0.134 | NA |
| *LOC114118629* | 2.050 | -0.134 | NA |
| *LOC114117059* | 1.996 | 0.388 | NA |
| *LOC114116764* | 1.973 | -0.634 | NA |
| *LOC114111821* | 1.915 | 0.887 | 0.067 |
| *LOC114115449* | 1.910 | 0.030 | NA |
| *LOC114110911* | 1.886 | 1.858 | NA |
| *LOC114116498* | 1.835 | -0.592 | 0.316 |
| *LOC114109491* | 1.834 | 0.720 | NA |
| *LOC114113369* | 1.823 | 0.272 | NA |
| *LOC114118120* | 1.823 | -0.917 | NA |
| *LOC114117371* | 1.818 | -0.270 | NA |
| *LOC114115452* | 1.818 | 0.521 | NA |
| *LOC114113400* | 1.808 | 0.521 | NA |
| *LOC114114641* | 1.808 | -0.135 | NA |
| *LOC114114258* | 1.807 | 0.775 | 0.044 |
| *LOC114111255* | 1.759 | -0.493 | NA |
| *LOC114110914* | 1.756 | 1.690 | NA |
| *LOC114114226* | 1.748 | -0.744 | NA |
| *LOC114117100* | 1.702 | -0.068 | NA |
| *LOC114117362* | 1.686 | 0.388 | 0.098 |
| *LOC114109223* | 1.675 | 1.090 | NA |
| *LOC114115448* | 1.663 | -0.613 | NA |
| *LOC114117366* | 1.661 | -1.087 | NA |
| *LOC114117061* | 1.628 | -0.744 | NA |
| *LOC114118596* | 1.591 | 0.062 | 0.805 |
| *LOC114114265* | 1.582 | -0.294 | NA |
| snRNA | | | |
| ID | VIP | log2FC | p-adj |
| *LOC114110889* | 2.612 | -0.040 | 0.992 |
| *LOC114108918* | 2.595 | -0.130 | 0.981 |
| *LOC114117103* | 2.486 | 2.063 | 0.961 |
| *LOC114117039* | 2.467 | 0.327 | 0.967 |
| *LOC114115467* | 2.325 | -1.255 | 0.961 |
| *LOC114108901* | 2.321 | 1.488 | 0.412 |
| *LOC114118780* | 2.314 | 1.489 | 0.412 |
| *LOC114110497* | 2.300 | -0.669 | 0.961 |
| *LOC114117414* | 2.273 | -0.276 | 0.961 |
| *LOC114109795* | 2.262 | 0.892 | 0.961 |
| *LOC114115133* | 2.260 | 0.202 | 0.975 |
| *LOC114108902* | 2.259 | 1.292 | 0.521 |
| *LOC114117027* | 2.254 | 0.392 | 0.967 |
| *LOC114111031* | 2.231 | 0.711 | 0.961 |
| *LOC114111020* | 2.231 | 0.711 | 0.961 |
| *LOC114111008* | 2.231 | 0.711 | 0.961 |
| *LOC114115136* | 2.231 | 0.711 | 0.961 |
| *LOC114117691* | 2.202 | 0.339 | 0.967 |
| *LOC114108924* | 2.202 | 0.339 | 0.967 |
| *LOC114110995* | 2.180 | -0.680 | 0.961 |
| *LOC114115966* | 2.173 | 0.424 | 0.967 |
| *LOC114114165* | 2.172 | 1.055 | 0.961 |
| *LOC114114674* | 2.172 | 0.490 | 0.967 |
| *LOC114117699* | 2.172 | 1.055 | 0.961 |
| *LOC114109457* | 2.170 | 0.903 | 0.961 |
| *LOC114118787* | 2.152 | -0.734 | 0.961 |
| *LOC114116739* | 2.135 | -0.610 | 0.961 |
| *LOC114115984* | 2.123 | -0.916 | 0.961 |
| *LOC114109272* | 2.102 | 0.964 | 0.961 |
| *LOC114111401* | 2.090 | -0.364 | 0.961 |

Top 30 potential noncoding RNA biomarkers for lncRNA, miRNA,snoRNA,snRNA comparing control male and T-treated male fetal LV tissue based on variable importance in Projection values along with the corresponding log2FC and padj values obtained from DESeq2 analysis for the same determinants are represented.
